# Engineering Proteins for Thermostability with iRDP Web Server

**DOI:** 10.1371/journal.pone.0139486

**Published:** 2015-10-05

**Authors:** Priyabrata Panigrahi, Manas Sule, Avinash Ghanate, Sureshkumar Ramasamy, C. G. Suresh

**Affiliations:** 1 Division of Biochemical Sciences, CSIR-National Chemical Laboratory, Pune, Maharashtra, 411008, India; 2 Division of Chemical Engineering and Process Development, CSIR-National Chemical Laboratory, Pune, Maharashtra, 411008, India; London, UNITED KINGDOM

## Abstract

Engineering protein molecules with desired structure and biological functions has been an elusive goal. Development of industrially viable proteins with improved properties such as stability, catalytic activity and altered specificity by modifying the structure of an existing protein has widely been targeted through rational protein engineering. Although a range of factors contributing to thermal stability have been identified and widely researched, the *in silico* implementation of these as strategies directed towards enhancement of protein stability has not yet been explored extensively. A wide range of structural analysis tools is currently available for *in silico* protein engineering. However these tools concentrate on only a limited number of factors or individual protein structures, resulting in cumbersome and time-consuming analysis. The iRDP web server presented here provides a unified platform comprising of iCAPS, iStability and iMutants modules. Each module addresses different facets of effective rational engineering of proteins aiming towards enhanced stability. While iCAPS aids in selection of target protein based on factors contributing to structural stability, iStability uniquely offers *in silico* implementation of known thermostabilization strategies in proteins for identification and stability prediction of potential stabilizing mutation sites. iMutants aims to assess mutants based on changes in local interaction network and degree of residue conservation at the mutation sites. Each module was validated using an extensively diverse dataset. The server is freely accessible at http://irdp.ncl.res.in and has no login requirements.

## Introduction

Thermophiles and hyperthermophiles are organisms that grow at extreme temperatures. Enzymes from these organisms are inherently stable and active at high temperatures, offering a major industrial advantage over their mesophilic homologues with respect to their storage, resistance against chemical denaturants and risk of microbial contaminations. Thermal stability is an important parameter that determines economic feasibility of applying an enzyme in any industrial process. Understanding the molecular determinants of thermostability can not only provide useful insights into evolution of such enzymes but the application of these through rational protein engineering to existing mesophilic proteins can also lead to development of more efficient and thermally stable biocatalysts for various industrial applications [1].

### Molecular determinants of protein thermostability

Studies have revealed several trends of residue preference towards thermostabilization of thermophilic proteins, such as lower content of uncharged polar residues, preference of arginine over lysine residues and higher charged residue contents [2]. Shortening of loop regions is a known mechanism of protein thermostabilization in hyperthermophilic proteins [3–5]. Various non-covalent interactions are known to play a vital role in thermostabilization of proteins [6–8]. Disulfide bridges are covalent interactions, which are known to provide stability to protein by entropic effect [9–11]. The presence of thermolabile residues and bonds involving asparagine and glutamine are known to introduce instability to the protein backbone by undergoing deamidation at elevated temperatures [12]. Residues in left-handed helical conformation upon mutation to glycine are known to contribute favorably to protein thermal stability [13, 14]. Marshall *et al*., 2002 have studied the interactions of α-helix dipole with side chains of sequentially charged residues and found it to contribute favorably to stability [15, 16]. Proline residues being conformationally most rigid are thought to provide stability to proteins by entropic effects [17, 18]. The hydrophobic effect is understood to be one of the primary driving forces of protein folding [19]. Decrease in hydrophobic surface area, as a stabilization mechanism has been studied in superoxide dismutase from *S*. *acidocaldarius* [20]. Bound metal is also vital for stability and functioning of many proteins [21–23].

### Target identification through comparative structural analysis

**Fig 1:**
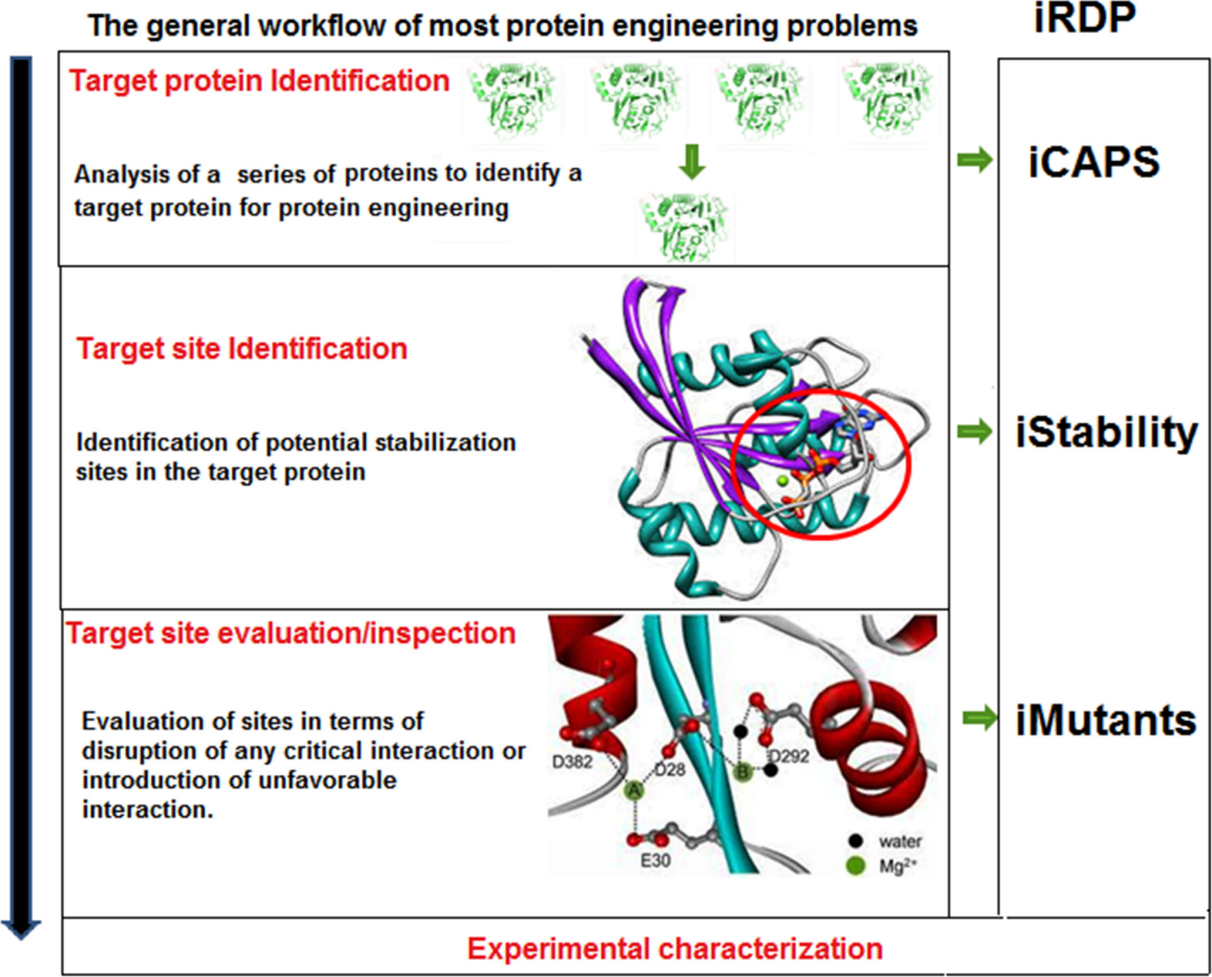
The general workflow of most rational protein engineering problems.

**Fig 2:**
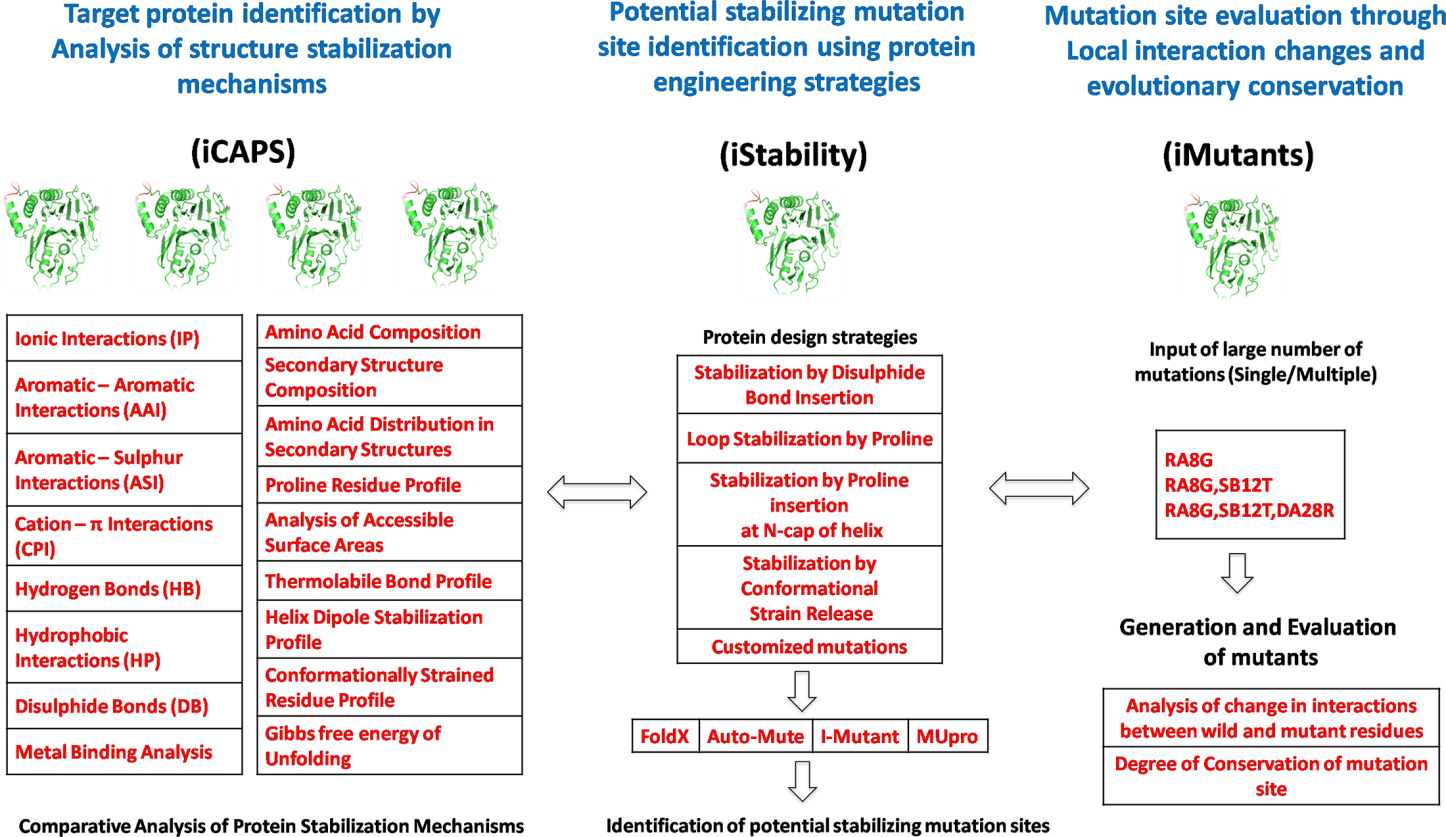
The three working modules of the iRDP web server implemented for rational engineering of proteins.

The rapid addition of protein structures to the PDB [24], has made manual analysis of combination of such a large number of factors extremely time-consuming and sometimes error-prone. Although a variety of computational tools such as WHAT IF [25], PIC [26] and Capture [27] are available for structural analysis, most of these are limited by their ability to analyse only a single structure at a time (S1 Table). These tools primarily focus on analysis of non-covalent interactions as stabilizing mechanisms ignoring most molecular determinants listed above. However, most protein engineering studies necessitate simultaneous analysis of several structural mechanisms amongst a vast set of protein structures for improved selection of target protein and potential mutation sites (Fig 1). In view of this, the iCAPS (***i*** *n silico* **C**omparative **A**nalysis of **P**rotein **S**tructures) module was developed to simplify the comparison process for a large number of protein structures in terms of the features listed above known to affect protein stability (Fig 2). iCAPS aims to help the user compare a series of proteins in order to select a target protein most suitable for initiation of protein engineering studies.

### Identification of potential sites for structural stabilization

Once a target protein is selected for engineering, the next task is to identify potential target mutation sites. The Suzuki group has conclusively proved entropic stabilization by proline insertion as a protein thermostabilization mechanism through their work on oligo 1,6 glucosidase from *Bacillus cereus*. Through this work, they were able to identify that proline insertion at second position of β-turns and N-cap of helix enhanced thermostability of the protein. This mechanism has been defined as the “The Proline Rule” [28, 29]. Loop stabilization by proline has been successfully implemented for thermostabilization of proteins such as cold shock protein, ubiquitin, ribonuclease Sa2 and guanyl specific ribonuclease Sa3, bacteriophage T4 lysozyme and human lysozyme [30–32]. Proline insertion at helix N-cap has also been used to enhance the stability of proteins like alcohol dehydrogenase, α-parvalbumin and triosephosphate isomerase [18, 33, 34]. Studies on ribonuclease HI show that release of conformational strain caused due to left-handed helical residue Lys95 upon mutation to Gly, results in considerable increase in thermostability of the protein [13]. This strategy has been used to improve the stability of proteins such as Drosophila adapter protein Drk, barnase, lysozyme and Pin1 WW [35–39]. In their classic work on bacteriophage T4 lysozyme, Matsumura *et al*., 1989, have not only elucidated the role of disulfide bridges in protein stability but have also shown that the effect of introduction of a combination of disulfide bridges on protein stability is additive in nature [9]. This mechanism has been used successfully for improving the stability of proteins like T4 lysozyme [40], subtilisin BPN [41], xylanase [42], lipase [43], lipase B [44] and glucose 1-dehydrogenase [45]. Currently computational tools such as CUPSAT [46], SDM [47], PopMusic [48], FoldX [49] and Rosetta Design [50] are available for predicting the effect of mutation on protein stability. However these tools require the user to input the mutations and do not suggest potential stabilizing mutations using specific strategies. Hence the iStability (***i*** *n silico* Analysis of **Stability** Change in Protein Structures) module was developed which not only aids in identification of stabilizing mutation sites through the application of protein design strategies described above for improvement of thermal stability but also assesses the stability of any mutant (Fig 2).

### Evaluating potential thermostabilization sites using molecular interactions

Once a stabilizing mutation site is selected, it is important to evaluate the mutation in terms of its effects on neighboring residues, which is vital to any protein engineering experiment. Serrano *et al*., 1992, in their work with barnase enzyme have revealed that loss of buried salt bridges and hydrogen bonds due to mutations affects protein stability significantly [37]. Studies carried out on the arc repressor protein of bacteriophage P22 have shown deleterious effects of mutations on protein stability due to disruptions in hydrogen bonds and salt bridges [51]. Computational tools such as CUPSAT, SDM, PopMusic and Rosetta Design (S2 Table) are mainly of predictive nature. Although, users are usually informed of the effects of mutations on protein stability by these tools in form of stability scores, underlying details of interaction rearrangements at the mutation site are currently not provided. Along with the stability scores, the information regarding the change in interactions could provide a better evaluation of the mutations being considered.

Understanding this, we have developed a mutation evaluation tool called iMutants (***i*** *n silico* Comparative Analysis of Interactions in Protein **Mutants**), which assesses the change in local interactions at mutation sites through comparison of wild-type and mutant proteins (Fig 2). Evolutionary conservation analysis is crucial to successful protein design since highly conserved positions typically play important structural or functional roles, i.e. mutation could adversely affect protein function [52]. Therefore iMutants also evaluates the conserved nature of the mutation sites.

The three modules, iCAPS, iStability and iMutants, addressing these aspects of protein engineering problems, are integrated on a single platform in the form of iRDP (***i*** *n silico* **R**ational **D**esigning of **P**roteins) web server presented in this work.

## Material and Methods

The iRDP server is built on a Linux platform using R, Perl, HTML and PHP. The Bio3d [53] and iGraph [54] packages form the core of all iRDP modules. The vast analysis carried out by modules of iRDP server uses, both in-house developed scripts and established tools (S3 Table). Described below is the detailed workflow of each module in iRDP web server (S1 Fig).

### *In silico* Comparative Analysis of Protein Structures (iCAPS)

Multiple structures serve as input to iCAPS. Input can be a list of PDB entries separated by commas, or files in valid PDB format can be uploaded. The user can select the structural features to be analyzed, modify cutoff values for calculation of non-covalent interactions and relative accessible surface area (ASA) before submitting the job. The relative ASA threshold is used to decide whether a residue is buried or exposed to solvent. The results page contains a unique job identification number for each job being submitted. Users can bookmark this page and return to view and retrieve the results later.

Analysis begins with primary structural features like amino acid composition, secondary structure content, information such as helix/strand/turn/coil composition and then proceeds to calculation of non-covalent interactions (Fig 2). The program DSSP [55] is used to detect secondary structures in the input proteins while NACCESS is used for estimation of residue solvent accessibility and accessible surface areas (ASA) [56]. The non-covalent interactions and disulfide bonds are identified using the standard criteria reported in literature (S4 Table). Users are provided with options to change the criteria of interaction calculations. In-house scripts are written for estimation of parameters such as proline residue distribution profile, thermolabile bond profile and helix dipole stabilization profile. Conformationally strained residues are identified by using Procheck [57] while β-turn and N-cap proline residues are identified using Promotif [58] and DSSP respectively. The program FindGeo has been employed for analysis of metal binding sites and geometry [59]. Gibbs free energy of unfolding is calculated using FoldX [49].

For the validation of iCAPS module, 16 thermophilic-mesophilic protein pairs were used (Table 1). Each pair was submitted to iCAPS module and raw values of various thermostability parameters were calculated. Raw values were first normalized by methods similar to that of Kumar *et al*., 2000 in order to estimate the percentage change of these parameters between mesophilic and thermophilic proteins [60]. Percentage change was calculated by using difference of normalized values between thermophilic and mesophilic protein, divided by the corresponding normalized value of mesophilic protein. For normalization of parameters such as total percentage of aromatic (Aro), uncharged polar (UP), proline (Pro), hydrophobic or aliphatic (ALI), charged (CHG) residues, total percentage of ion-pairs (IP), aromatic-aromatic (AAI), aromatic-sulphur (ASI), cation-pi (CPI), hydrogen bonding (HB), hydrophobic (HP) interactions, total percentage of conformationally strained residues (CS) and percentage of residues in loop regions (Loop), the raw values obtained were normalized using sequence length. In case of parameters such as total percentage of proline residues occurring at 2^nd^ position of beta turns (Bt2P) and Ncap helix positions (NCap), normalization was carried out using total number of proline residues. Similarly the total percentage of dipole-stabilized helices parameter was normalized with total number of helices. In case of normalization for total percentage of thermolabile bonds (TL), raw values were normalized using total number of Asn and Gln residues. For normalization of parameters such as ratio of Nonpolar to Polar accessible surface areas (NP/P) and Arg to Lys ratio (R/K), raw values were directly considered for percentage change calculation. Since the protein families considered for analysis in the dataset were highly diverse in terms of sequence and structure, the normalization process focused on the pairs rather than the entire dataset.

**Table 1:** Comparative analysis of various thermostability factors among 16 thermophilic-mesophilic pairs of protein.

	Thermophilic (TS) and Mesophilic (MS) protein pairs**																Total positive values***
TS*	1AJ8	1BDM	1CAA	1CIU	1GTM	1LDN	1LNF	1PHP	1TMY	1XGS	1YNA	1ZIN	1IQZ	2PRD	3MDS	3PFK	
MS*	1CSH	4MDH	8RXN	1CDG	1HRD	1LDG	1NPC	1QPG	3CHY	1MAT	1XNB	1AKY	1FCA	1INO	1QNM	2PFK	
**Aro**	0.17	-0.03	-0.02	0.15	-0.05	0.81	0.11	-0.06	-0.41	0.25	-0.05	0.32	1.38	-0.18	0.16	-0.22	8
**UP**	-0.37	-0.23	-0.22	0.12	-0.09	-0.19	-0.09	-0.39	-0.21	-0.28	-0.28	-0.26	-0.22	-0.28	-0.26	0.17	2
**Pro**	-0.17	0.26	-0.18	-0.10	0.02	-0.25	0.34	-0.07	0.78	0.14	-0.05	-0.30	0.02	-0.15	0.27	-0.12	7
**ALI**	0.09	-0.03	0.12	0.05	0.04	-0.14	0.14	0.06	0.12	0.05	0.03	0.11	0.05	0.10	0.07	-0.07	13
**CHG**	0.32	-0.05	0.26	-0.05	0.15	0.10	0.04	0.12	0.10	-0.01	0.45	0.01	0.89	0.01	0.07	0.01	13
**R/K**	-0.38	2.50	#	0.26	-0.07	4.26	0.60	1.14	-0.19	-0.25	0.91	1.83	#	1.28	0.59	-0.32	9
**IP**	0.54	-0.01	5.87	-0.01	0.57	0.20	0.00	0.70	0.51	0.45	1.45	-0.12	3.07	0.08	-0.27	0.50	11
**AAI**	0.24	0.92	-0.02	0.00	-0.20	6.50	0.06	0.05	-3.13	0.79	-0.05	0.92	0.00	0.01	-0.37	-0.16	8
**ASI**	-0.61	5.13	-0.02	0.23	-0.54	0.00	-0.32	2.16	0.07	-0.85	-0.52	0.92	0.02	-0.50	-0.51	-0.33	6
**CPI**	0.94	-0.42	0.23	0.07	-0.45	6.00	0.08	0.05	-0.47	-0.62	-0.15	-0.32	3.07	-0.06	-0.21	0.94	8
**HB**	-0.05	0.10	0.16	0.03	0.08	-0.14	0.00	0.06	-0.04	0.01	0.04	0.09	0.58	0.14	0.01	0.05	12
**HP**	0.17	0.16	0.01	0.01	0.11	0.10	0.16	0.08	-0.06	0.47	-0.09	-0.29	-0.16	-0.06	0.23	-0.11	10
**Bt2P**	-0.65	-0.19	-0.20	0.12	-0.40	0.33	0.50	-1.00	-0.40	-0.61	0.00	0.44	0.00	0.48	-0.49	0.50	6
**NCap**	-0.19	~	0.20	-0.72	1.11	0.33	-0.25	0.51	~	-1.00	#	-0.04	#	#	~	-0.50	7
**Hdip**	-0.11	0.03	0.00	0.26	0.23	0.01	-0.20	0.06	-0.17	0.07	0.00	-0.11	0.33	0.25	-0.27	-0.07	8
**TL**	-0.04	0.83	0.00	0.05	0.01	0.89	-0.30	-0.39	1.86	0.39	-0.15	-0.07	-0.50	-1.00	0.26	1.17	8
**CS**	1.35	-0.23	#	-0.11	-0.25	-0.33	-0.14	-0.12	~	-0.40	-0.05	-1.00	-0.15	-0.50	#	-0.14	2
**NP/P**	0.00	-0.08	0.00	-0.07	-0.01	-0.15	-0.10	-0.09	0.16	-0.22	-0.04	-0.25	-0.16	-0.11	0.01	0.01	3
**Loop**	0.09	-0.12	-0.05	0.07	-0.01	0.06	0.08	-0.01	-0.22	-0.12	-0.03	-0.05	-0.12	-0.17	0.15	0.10	6

* The PDB IDs of Thermophilic (TS), Mesophilic (MS) pair, starting from Column 2, belong to family Citrate Synthase, Malate dehydrogenase, Rubredoxin, Cyclodextrin, Glutamate dehydrogenase, L-Lactate dehydrogenase, Thermolysin, 3-Phosphoglycerate kinase, Chey protein, Methionine aminopeptidase, Endo–1,4-Beta-Xylanase, Adenylate kinase, Ferredoxin, Pyrophosphate phosphohydrolase, Manganese superoxide dismutase and Phosphofructokinase. The parameters listed in Column 1 correspond to aromatic (Aro: residues FWY), uncharged polar (UP: residues NQST), proline (Pro), hydrophobic or aliphatic (ALI: residues VILM), charged (CHG: residues DERKH) residue contents, Arg to Lys ratio (R/K), total percentage of ion-pairs (IP), aromatic-aromatic (AAI), aromatic-sulphur (ASI), cation-pi (CPI), hydrogen bonding (HB), hydrophobic (HP) interactions, proline residue percentages occurring at 2^nd^ position of beta turns (Bt2P) and Ncap helix positions (NCap), percentage of dipole stabilized helices (Hdip), thermolabile bonds (TL) and conformationally strained residues (CS), ratio of nonpolar to polar accessible surface areas (NP/P) and percentage of loop region (Loop).

**The value shown in # represents the case in which both MS and TS proteins show absence of the corresponding parameters while the values shown in ~ represents the case in which only the MS protein shows absence of the corresponding features.

*** Total number of positive values calculated for each parameter; indicates number of families in which thermophilic proteins have higher preference for the parameter than their mesophilic counterparts. Numbers of ~ values are also considered while counting total number of positive values. Detailed results can be found at http://irdp.ncl.res.in/cgi-bin/result_fetch.php?ID=iCAPScase.

### *In silico* Analysis of Stability Change in Protein Structures (iStability)

The input to this module consists of providing a PDB code or uploading a valid PDB formatted file. The user must select any of the pre-defined protein design strategies (Fig 2) or provide their own mutations in the specified format. Once the design strategy is selected, the user has the choice of trying out four different stability prediction tools (FoldX [49], Auto-Mute [61], I-Mutant [62] and MUpro [63]), which are based on empirical potential energy functions or machine learning methods with provision to modify input. In case of FoldX, mutations are carried out and the total energy difference between the mutant and the corresponding wild-type is estimated. Positive values of energy difference reflects less stability. Auto-mute uses machine learning approach to carry out computational mutagenesis. A 3D-1D profile is generated for both the mutant and wild-type, which is further used in the generation of a vector difference profile. Here the environmental change (EC) scores quantify the differences generated for the mutant in terms of confidence in stability classification. I-Mutant implements support vector machines based on potential energy function to determine the stability. The ΔΔG value calculated uses the difference of Gibbs free energy (ΔG) of the mutant and wild-type. If the ΔΔG value is < 0 kcal/mol, the mutant is considered to be less stable than wild-type, while if the ΔΔG value is > 0 kcal/mol, the mutant is said to be more stable then wild-type. MUpro predicts how single-site amino acid mutations affect protein stability using the ΔΔG value, which is computed using support vector machines and neural networks. The confidence score generated measures the confidence of the prediction. If the score is < 0, it indicates decreased stability, whereas, score > 0 shows that the mutation increases stability compared to wild-type. The user can choose residue conservation analysis if required. If input is a PDB entry, then the evolutionary residue conservation score is derived from ConSurf-DB [64], on a scale of 1–9 (1 is an indication of least conserved/highly variable and 9 highly conserved/least variable). If input is a structure uploaded by the user, then the extracted sequence is used to search for homologs against the UniRef90 database using PSI-BLAST (2 iterations and e-value cutoff of 1) [65]. Weighted observed percentages from generated Position Specific Scoring Matrix (PSSM) are scaled from 1 to 9 as already defined and are presented as conservation scores.

For validation of iStability module, a total of 17, 6, 10 and 15 proteins were selected for the analysis of beta-turn proline insertion, N-cap proline insertion, conformational strain release and disulfide bond insertion strategies respectively (Table 2 and S7 Table). Protein structures were analyzed using iStability module by selecting each strategy and predictions obtained were compared with experimental observations.

**Table 2:** Validation of iStability using beta-turn proline insertion and conformational strain release strategy.

PDB ID	Protein	Organisms	Mutation	Experiment*	iStability**	FoldX energy (kcal/mol)**	Reference
**Stabilization by insertion of Proline residues at 2** ^**nd**^ **position of Beta-turns**							
1CSP	Cold shock protein	*Bacillus subtilis*	N55P	I (1.0 kcal/mol)	I	-0.34	[30]
1ZW7	Ubiquitin	*Saccharomyces cerevisiae*	S19P	I (0.9 kcal/mol)	I	-1.72	
1PYL	Ribonuclease Sa2	*Streptomyces aureofaciens*	N33P	I (0.5 kcal/mol)	I	-1.24	
			N51P	I (0.7 kcal/mol)	I	-0.53	
1MGR	Guanyl-specific ribonuclease Sa3	*Streptomyces aureofaciens*	S34P	I (0.9 kcal/mol)	I	-1.33	
			T52P	I (0.5 kcal/mol)	I	-0.52	
9RNT	Ribonuclease T1	*Aspergillus oryzae*	S63P	I (0.8 kcal/mol)	I	-2.28	
2RN2	RibonucleaseH	*Escherichia coli*	A93P	N (-0.1 kcal/mol)	I	-1.13	
			G123P	I (0.3 kcal/mol)	I	-1.84	
3MBP	Maltose Binding Protein	*Escherichia coli*	G13P	N (0 kcal/mol)	I	-1.99	
			A206P	N (-0.1 kcal/mol)	I	-2.17	
1RGG	Ribonuclease (RNase) Sa	*Streptomyces aureofaciens*	S31P	I (0.7 kcal/mol)	I	-1.28	[72]
			T76P	I (1 kcal/mol)	I	-0.89	
1PGA	Protein G	*Streptococcus sp*. *GX7805*	K10P	D (-8.4°C)	D	0.31	[73]
			A48P	D (-6.8°C)	I	-0.52	
2LZM	Bacteriophage T4 Lysozyme	*Enterobacteria phage T4*	A82P	I (0.8°C)	I	-1.35	[32]
1UOK	Oligo–1, 6-glucosidase	*Bacillus cereus*	K121P	I (4.6 kJ/mol)	I	-0.36	[17]
			E208P	I (11.7 kJ/mol)	I	-1.35	
			E290P	I	I	-0.85	
2IMM	IgA-Kappa MCPC603 FV (Light chain)	*Mus musculus*	A15P	I	I	-1.37	[74]
			S56P	I	I	-0.69	
			D60P	I	I	-1.14	
			G68P	D	D	6.77	
1LZ1	Lysozyme	*Homo sapiens*	A47P	I (0.3°C)	I	-1.37	[31]
1KEV	Alcohol dehydrogenase	*Clostridium beijerinckii*	S24P	I (3.9°C)	I	-1.56	[18]
1LVE	Immunoglobulin K–4 light chain Len	*Homo sapiens*	L15P	D (-1.15 kcal/mol)	I	-0.22	[75]
1RTP	Alpha-Paravalbumin	*Rattus rattus*	A21P	D (-8.5°C)	I	-0.96	[33]
3GLY	Glucoamylase	*Aspergillus awamori*	S30P	I (1.6 kJ/mol)	I	-1.27	[76]
**Stabilization by Conformational Strain release strategy**							
1A5E	Cyclin-dependent kinase inhibitor	*Homo sapiens*	L78G	I (0.9°C)	I	-2.68	[77]
1LZ1	Lysozyme	*Homo sapiens*	R50G	I (0.9°C)	I	-1.2	[38]
			Q58G	I (5.7°C)	I	-0.58	
			R21G	I (3.7°C)	D	0.47	[39]
			N118G	I (0.2°C)	I	-0.47	
1PIN	Pin1 WW domain	*Homo sapiens*	N30G	I (6.4°C)	D	0.51	[78]
			S18G	I (0.02 kcal/mol)	I	-1.14	
1STN	Staphylococcal nuclease	*Staphylococcus aureus*	K136G	I (0.1 kcal/mol)	D	0.99	[79]
2AFG	Acidic fibroblast growth factor	*Homo sapiens*	N106G	I (0.38 kcal/mol)	I	-0.4	[80]
2RN2	Ribonuclease HI	*Escherichia coli*	K95G	I (5.7°C)	I	-1.51	[13]
1BNI	Barnase	*Bacillus amyloliquefaciens*	H18G	I (0.51 kcal/mol)	I	-1.59	[81]
2AFG	Acidic fibroblast growth factor	*Homo sapiens*	H93G	I (1.32 kcal/mol)	I	-1.59	[82]
1ROP	Rop	*Escherichia coli*	D30G	I (11.6°C)	I	-1.58	[83]
2A36	Drk	*Drosophila melanogaster*	T22G	I (3.6 kcal/mol)	I	-3.71	[35]

* The labels I, D and N correspond to an increase, decrease and no change in stability respectively for the mutations as inferred from experiment. The values with unit kcal/mol represent ddG value (Change in free energy of unfolding, Mutant—Wild-type) while those with unit°C represent dTm value (Change in midpoint temperature of the thermal unfolding, Mutant—Wild-type) as inferred from the experiment. A positive value represents an increase in stability.

**Two states of iStability predictions considered are: I (FoldX energy < 0) representing increased stability and D (FoldX energy > 0) representing decreased stability.

### *in silico* Comparative Analysis of Interactions in Protein Mutants (iMutants)

iMutants takes a single structure as input similar to iStability. The user must also provide mutations in the specified format. A large number of mutations can be analyzed simultaneously in iMutants. Each mutation must be provided in a separate line. For double or multiple mutants, mutations should be provided in a comma-separated format on a single line (S2 Fig). Users can modify interaction cutoffs and relative ASA value before submitting a job. Similar to iStability, an option is provided for residue conservation analysis at the mutation site.

For validation of iMutants module, a total of 51 mutations were analyzed (Table 3 and S8 Table) in arc repressor protein of bacteriophage P22 (PDB ID: 1ARR). Mutants were generated using MODELLER [66], energy minimized using steepest descent and finally the structure thus generated was compared with wild-type structure to analyze the change in interactions at the mutation site.

**Table 3:** iMutant analysis for the five highly unstable mutations in Arc repressor protein.

MutNo	Type	Chain	Res No	ResID	Local Interaction Profile*										
					IP	IPNet	AP	APNet	AS	ASNet	HB	Disul	Catpi	CatpiNet	Hphob
1	wild	A	22	V	-	-	-	-	-	-	2	-	-	-	7
	mut	A	22	A	-	-	-	-	-	-	2	-	-	-	2
2	wild	A	37	I	-	-	-	-	-	-	3	-	-	-	9
	mut	A	37	A	-	-	-	-	-	-	2	-	-	-	3
3	wild	A	41	V	-	-	-	-	-	-	2	-	-	-	6
	mut	A	41	A	-	-	-	-	-	-	1	-	-	-	3
4	wild	A	45	F	-	-	-	-	-	-	-	-	-	-	6
	mut	A	45	A	-	-	-	-	-	-	-	-	-	-	2
5	wild	A	36	E	2	1	-	-	-	-	2	-	-	-	-
	mut	A	36	A	-	-	-	-	-	-	1	-	-	-	2

*The labels in local interaction profile correspond to the number of IP: Ion-pair, IPNet: Ion-pair networks, AP: Aromatic-aromatic interaction, APNet: Aromatic-aromatic interaction network, AS: Aromatic-sulphur interactions, ASNet: Aromatic-sulphur interaction network, HB: Hydrogen bonds, Disul: Disulfide bonds, Catpi: Cation-pi interactions, CatpiNet: Cation-pi interaction networks and Hphob: Hydrophobic interactions, formed by wild-type (wild) and mutant (mut) residues. The—(hyphen) corresponds to no interaction or interaction networks detected. Please refer S8 Table to see the contribution of other interactions.

## Results and Discussion

The generalized workflow that can be implemented for most rational protein engineering problems has been described in Fig 1. The features and *in silico* implementation of this workflow in iRDP web server has been depicted in Fig 2.

### Description and Validation of the Modules

#### Analysis of structure stabilization mechanisms using iCAPS

iCAPS has been designed to carry out a comparative analysis of protein structures in terms of structural features and interactions that are known to contribute to thermodynamic stability. iCAPS supports investigation of 20 different stabilization mechanisms, as described below, estimating more than 250 parameters (S4 Table) analyzed simultaneously for a maximum of 100 structures.

1. **Amino acid composition**: iCAPS generates a comparative summary for a set of proteins in terms of their amino acid composition and its property-wise classification into different categories like positively charged, negatively charged, uncharged polar and aromatic residues.
2. **Secondary structure information**: Comparative summary generated for overall secondary structure (SS) content as well as the residue composition of each type of SS (Helix/Strand/Turn/Coil) of proteins, provides better understanding of the contribution of SS to protein thermostabilization.
3. **Non-covalent interactions**: iCAPS calculates non-covalent interactions such as ion-pairs (IP), aromatic-aromatic interactions (AAI), aromatic-sulphur interactions (ASI), cation-π interactions (CPI), hydrogen bonds (HB) and hydrophobic interactions (HP). It also offers identification of interaction networks that are energetically more favorable compared to isolated interactions.
4. **Disulfide bridges**: iCAPS identifies disulfide bridges in input protein structures and provides useful insights by classifying them in terms of their expected entropic effect (i.e. based on the number of residues between bridged Cys residues) while providing other details such as solvent accessibility and SS preference of Cys residues, revealing the contribution of these bonds to structural stabilization.
5. **Thermolabile bond profile**: iCAPS studies spatial distributions of thermolabile bonds as potential target sites for stability enhancement in input structures involving asparagine and glutamine along with additional details such as SS preference and solvent accessible nature of the residues forming these bonds.
6. **Conformationally strained residue profile**: Conformationally strained residue detection feature in iCAPS identifies conformationally strained residues in input structures, which could be considered for mutation to glycine for improving thermal stability of proteins. The module also provides additional information such as conformational geometry, SS preference and strain distance (distance between the C_β_ and main chain oxygen atom) of the strained residues. While mutation of such residues to Gly remain the most established strategy, the void generated due to the lack of side chain in the Gly residue remains a viable concern [67]. In such cases it is advisable to explore non-Glycine substitutions using the customized mutation option in iStability.
7. **Helix dipole stabilization profile**: The helix dipole stabilization feature of iCAPS identifies dipole-stabilized helices in input protein structures along with position-wise classification of dipole stabilizing charged residues.
8. **Proline residue profile**: iCAPS reports the distribution of proline residues in various secondary structures along with specific identification of prolines occurring at the second position of beta-turns and at N-terminus of helices. Since the solvent exposed loops and intrinsically disordered regions of proteins are often found to be proline-rich [68], the secondary structure wise proline distribution provided warrants careful analysis.
9. **Accessible Surface Area analysis**: iCAPS measures the total, main-chain, side-chain, polar and non-polar accessible surface areas of proteins. ASA analysis also classifies all 20 amino acids by their solvent accessible nature as buried or exposed.
10. **Metal binding analysis**: The module identifies residues involved in metal binding sites along-with determination of metal coordination geometries.
11. **Estimation of Gibbs free energy of unfolding**: Protein stabilization energies for input structures are computed by iCAPS using the FoldX energy function [49]. The total energy is considered as an approximation of overall stability of the protein. This comparative report gives a comprehensive overview of energies involving various structure stabilization mechanisms amongst proteins under study.

The above results are presented in a formatted web page. Besides this, for further down-stream analysis the user can download a zipped file containing all results in the form of tab-delimited text files. An extensive help file has been prepared and provided in the website which explains the importance of every parameter generated, along with relevant references.

#### Validation of iCAPS module

For validation of iCAPS, a diverse non-redundant dataset of thermophilic-mesophilic (TS-MS) protein pairs, from organisms that are moderately thermophilic to hyperthermophilic as well as their mesophilic counterparts, were investigated (Table 1). The pairs comprising of structures having resolution ≤ 2.5 Å were selected from a diverse set of families (S5 Table). The selected TS-MS pairs were observed to be highly similar to each other with RMSD of the structures in the range of 0.69–1.68 Å while sequence identity in the range of 24–73% (S3 Fig). The thermophilic and mesophilic protein set among themselves were found to be highly dissimilar with the sequence identity ranging from 1–12% and 2–13% respectively (S3 Fig), demonstrating the diverse families considered for the analysis. In terms of structural diversity, 2 families were found to belong to all-alpha class, 3 to all beta class, 1 belonging to small proteins while the rest belonged to alpha-beta class according to the SCOP classification [69]. In most cases the oligomeric state of the pairs selected was found to be the same. Table 1 shows the percentage change values between TS-MS pairs with respect to various structural parameters estimated by iCAPS. The percentage change values can be correlated to the extent of the contribution of each of the factor towards thermostability of the proteins in the dataset. A positive value indicates higher occurrence of a particular parameter in thermophilic proteins while a negative value corresponds to higher occurrence in their mesophilic counterparts.

Comparative amino acid composition analysis revealed 13 families showing higher preference of charged (CHG) amino acids while 14 families displayed a lesser content of uncharged polar amino acids (UP) in the thermophilic proteins (Table 1). Of the 16 families, Ferredoxin from *Bacillus thermoproteolyticus* (1IQZ) was observed to show highest preference for charged residue content compared to its mesophilic partner from *Clostridium acidurici*. Similarly 3-Phosphoglycerate kinase from *Geobacillus stearothermophilus* (1PHP) showed lowest preference for UP content compared to its mesophilic homolog from *Saccharomyces cerevisiae*. This preference for charged residues compared to uncharged polar residues, a thermostabilization trend [70], also affected other parameters such as ion-pairs (IP), and the R/K ratio. It was found that 11 families had higher numbers of ion-pairs. The percentage change of ion-pairs was observed to be highest in case of Rubredoxin, a 53-residue protein. Rubredoxin from thermophilic *Pyrococcus furiosus* (1CAA), has 7 ion-pairs in its structure (5 ion-pairs form a network) while its mesophilic counterpart from *Desulfovibrio vulgaris* showed only one ion-pair. Similarly, 9 thermophilic proteins showed higher preference for Arginine than Lysine with highest preference observed in case of L-Lactate dehydrogenase enzyme family (1LDN). In terms of hydrogen bonding interactions (HB), 13 families contained a higher number of hydrogen bonds in the thermophilic set as compared to their mesophilic counterparts, thereby revealing hydrogen bonds as a contributing factor towards better protein stability. For this dataset aromatic residue content (Aro) and interactions involving aromatic amino acids (AAI, ASI and CPI) showed lower contribution towards stability. Hydrophobic residue content (ALI) and hydrophobic interactions (HP) were observed to be higher in 13 and 10 families of thermophilic proteins respectively. Among all pairs in the dataset, Methionine aminopeptidase from *Pyrococcus furiosus* (1XGS) showed highest hydrophobic interactions compared to its mesophilic counterpart from *Escherichia coli*. Reduction in hydrophobic surface area of a protein is a known thermostabilization mechanism. It was seen that in 11 cases the change in NP/P ratio was found to be negative (highest in case of Adenylate kinase family). While 7 families in the thermophilic set showed higher Pro content, in the current dataset only 6 and 7 thermophilic proteins respectively show beta-turn (Bt2P), NCap proline insertion parameters to be a contributing factor. Contribution by shortening of loop regions (Loop) in proteins towards thermostability was observed in 10 thermophilic proteins. In case of CheY protein family, loop percentage was observed to be lowest in case of thermophilic protein (1TMY) than its mesophilic partner. It was similarly observed that 12 thermophilic proteins contained fewer conformationally strained residues (CS), a factor contributing positively towards thermostability.

Although it was difficult to observe a generalized rule for protein thermostabilization, the analysis highlighted few parameters such as charged residue preference, increased ion-pairs and hydrogen bonding interactions, decreased non-polar accessible surface area, conformationally strained residues, and shortening of loops to contribute positively to thermostability of proteins in this dataset.

#### Identification of potential stabilizing mutations in a protein using iStability

iStability offers *in silico* implementation of four experimentally established protein engineering strategies (Fig 2) for identification of possible stabilizing mutation sites. iStability also allows for analysis of any other structure stabilization mechanism based on user-defined mutations. Evolutionary conservation scores further help to decide the mutability of mutation sites, highly conserved sites should be considered for mutation with caution. For further downstream analysis, generated mutant structures can be downloaded. The identification of mutation sites along with stability predictions and residue conservation makes iStability a unique *in silico* protein-engineering tool.

For the insertion of disulfide bonds strategy, iStability invokes SSBOND software [71], which identifies and ranks residue pairs that on mutation to cysteines could form stable disulfide bridges. Based on identified residue pairs, disulfide bonds are inserted and their effect on stability is predicted. In case of entropic stabilization strategy by insertion of proline residues, iStability identifies the beta-turns in the protein containing non-proline residues at second position and helices containing non-proline residues at the N-cap position. Based on the stability prediction tool (Fig 2) selected, residues in the identified positions are mutated to proline. The mutant structures are then compared with the wild-type by the tool in order to generate a stability score. This stability score is then used to report if the stability of the mutant has been found to increase (I) or decrease (D) in comparison to the wild type. Similarly for the release of conformational strain strategy, iStability identifies conformationally strained residues, mutates them to glycine to release the strain and predicts their effects on stability. Apart from implementation of these strategies, iStability reads user-defined mutations through the customized mutation feature and predicts the mutant stability. The results of iStability constitute the stability score, stability prediction (I: increasing stability and D: decreasing stability) and conservation score of the residue being mutated.

#### Validation of iStability module

For the validation of iStability module, a total of 81 mutations exploring the strategies implemented, from a diverse set of 40 structures (S6 Table) were analyzed for their stability effect, by selecting FoldX as stability prediction tool.

i. Improvement of protein thermostability by entropic reduction due to Proline introduction

Using iStability with default parameters, we have studied 28, second position β-turn proline insertions in 17 proteins from 13 organisms for which experimental stability results were available (Table 2). Of 28, iStability could accurately predict the stabilities for 22 β-turn insertions. In 20 cases, upon proline insertion an increase in stability was observed both experimentally and in iStability results. In the case of Protein G from *Streptococcus sp*. *GX7805*, the mutation K10P was predicted to decrease the stability by iStability which correlated with experimental results showing a decrease of 8.4°C in the T_m_ value of the mutant. For the G68P mutation in 2IMM, experimentally a significant decrease in stability was observed which iStability also predicted accurately. For three cases (A93P in 2RN2, G13P and A206P in 3MBP) showing near wild-type stability experimentally, iStability predicted an increase in stability. For 3 other cases (A48P in 1PGA, L15P in 1LVE and A21P in 1RTP), the module was unable to predict the stability of the mutants correctly as experimentally they were observed to have decreased stability whereas iStability predicted them to have increased stability.

11 proline insertions at the N-cap position of helices were analysed in 6 proteins from 5 organisms (S7 Table) for which experimentally determined stability results were available. Of the 11 mutations only 1 mutation, namely L316P carried out for Alcohol dehydrogenase was predicted inaccurately by iStability. The experimental results [18] for this mutant indicate the mutant to have higher stability (ΔT_m_: +10.8°C) than wild-type while iStability predicts a decreased stability for the mutant.

ii. Increasing protein thermostability through release of conformational strain by mutation to Glycine

A total 14 conformational strained residues in 10 proteins from 5 organisms (Table 2) were studied using iStability and the predictions were compared with experimentally validated results. The stability of 11 mutants was predicted accurately by iStability. Only 3 cases (R21G in 1LZ1, N30G in 1PIN and K136G in 1STN) were predicted incorrectly by the iStability module. Experimentally these three mutants were found to be thermostable [36, 39, 79] while iStability prediction shows a decreased stability.

iii. Reducing entropy for enhancement of thermostability by introduction of disulfide bridges

A set of 28 double Cysteine mutations (S7 Table) was studied for insertion of disulfide bonds for enhancement of protein stability in 15 proteins from 9 organisms. For this strategy, though iStability detected all the 28 residues pairs as potential insertion sites, stability was predicted correctly in 11 cases in comparison with experimentally determined stabilities. In one case (T72C, A471C in 3GLY), experimental evidence showed near wild-type stability while iStability predicted increased stability.

Of the total 81 predictions studied, 47 were true-positives (Both experiment and predictions showed increase of stability), 8 were true-negatives (Both experiment and predictions showed decrease of stability), 9 were false-positives (Experiment showed decrease while prediction showed increase of stability) and 17 were false-negative (Experiment showed increase while prediction showed decrease of stability). Thus the true-positive rate calculated was 0.84 while false-positive rate was 0.53. By using different FoldX energy cut-offs, neutral-states were incorporated in the prediction and ROC curve was generated (S9 Table and S4 Fig). While the true-positive rate and false-positive rates calculated actually test the accuracy of the underlying stability prediction program selected (FoldX), the values shown above also reflect the importance of using protein design strategies for better prediction of mutation sites. In those cases where iStability prediction differed from that observed experimentally, further analysis was carried out using iMutants. In case of A48P, the beta-turn proline insertion strategy in 1PGA, iMutants analysis revealed the loss of A48 (N)–(OD1) 46D hydrogen bond in mutant protein. This loss of hydrogen bond could result in decrease in stability observed experimentally. Similar changes in interactions near the mutation site were also noted in other cases, suggesting the need for further evaluation of the identified mutants using iMutants.

iStability currently relies on use of stability prediction tools that are based either on empirical potential energy functions or machine learning methods. Since these tools are based on defined training dataset, predicting mutations that are distant to the training dataset are a cause for concern.

#### Evaluating mutations through interaction framework and evolutionary residue conservation at mutation sites using iMutants

A comparative local interaction profile generated from the detailed molecular interactions in a model is unique to iMutants. It offers a quantitative measure of structural changes in mutants, through loss or gain of interactions at the mutation site. The module provides a comparative interaction analysis of wild-type and mutant residues summarized in the form of a local interaction profile comprising a number of interactions and their networks. Hyperlinks provide details of the calculated interactions. iMutants also supplements the interaction profile with estimated evolutionary conservation scores of the wild-type residues being mutated. In addition, mutant structures generated can also be downloaded for further downstream analysis.

#### Validation of iMutants module

The equilibrium stabilities of 51 mutants for the arc repressor protein of bacteriophage P22 (PDB ID: 1ARR) have been studied experimentally by Milla *et al*., 1994, using thermal and urea denaturation [51]. These 51 mutations were analysed using the iMutants module and the change in various non-bonded interactions was recorded. The mutations were divided into four groups as established by Milla *et al*., 1994 for analysis purposes. The first group consisted of 5 mutants (Table 3; V22A, I37A, V41A, F45A, E36A) that were experimentally determined to be highly unstable as they were unable to form dimers, and remained in an unfolded state. Of the five, the first four mutations showed a dramatic decrease of 5, 6, 3 and 4 hydrophobic interactions, respectively. These interactions affect the hydrophobic core of the protein and the loss of interactions coincides with the experimental instability observed. Since the E36A mutation involves alteration of a buried polar residue, iMutants recorded drastic loss of two ionic interactions, one ionic network along with one hydrogen bond, which was established by Milla *et al*., 1994 as a possible cause of instability of the mutant.

The next set analysed comprised 20 mutants (S8 Table), which experimentally exhibited reduced stability with t_m_ values ranging from 30–50°C as compared to the wild-type protein (T_m_: 57.9°C). Since three mutations R31A, R40A and R50A involved polar residues, iMutants recorded a loss of one ionic interaction and one ionic interaction network for R31A, two ionic interactions, two hydrogen bonds and one ionic interaction network for R40A and finally one ionic interaction for R50A mutants that could explain the instability of these mutants (T_m_: 37.1°C, 31.2°C and 47.9°C, respectively). For mutants W14A, L21A, N29A, V33A and Y38A, changes in hydrogen bonding interactions were observed (S8 Table). The mutation W14A also showed a loss of two aromatic pair interactions, one aromatic pair network as well as five hydrophobic interactions. This change in interactions could thus explain the decrease of T_m_ to 31.5°C observed for this mutant. Mutants F10A, L12A, P15A, L19A, L21A, Y38A and M42A showed a loss of 6, 4, 2, 4, 1, 3, and 2 hydrophobic interactions respectively which could contribute to the instability observed.

The set of 25 mutants (S8 Table) analysed next displayed near wild-type stability experimentally with their t_m_ ranging between 55–63°C. Most mutations in this set showed marginal or no change in their hydrogen bonding, ionic and hydrophobic interactions. P8A, the only mutant with increased stability (T_m_: 74.1°C), showed a change in just one hydrophobic interaction in the iMutants analysis (S8 Table). The stabilization of this particular mutant could be due to the extension of β-sheets or relief of unfavourable packing interactions as postulated by Milla *et al*, 1994.

In summary, hydrogen bonds and hydrophobic interactions were observed to provide a sizable contribution towards stability of arc repressor protein. Complete interaction profiles and details of interactions for mutants along with their T_m_ values have been provided in S8 Table. Although the experimental evidence by Milla *et al*., 1994 focus solely on mutation of the residues to Ala, exploring mutations to non-Ala residues could also yield additional useful information.

#### iATMs (*in silico* Analysis of Thermally stable Mutants): An information resource

The local interaction analysis approach of iMutants was extended to analyse experimentally validated mutations listed in the ProTherm database and is provided in the form of iATMs (***i*** *n silico* **A**nalysis of **T**hermally stable **M**utants), as a supplementary information resource to ProTherm [84]. Although ProTherm contains a vast resource of experimental information, no information is available describing the changes in structure and atomic interactions due to the mutations carried out. iATMs is organized in three sections based on the type of mutation as single, double or multiple. Within these, the sections are further classified into those containing crystal structures for both wild-type and mutant proteins and those where only wild-type crystal structures are available. Wherever wild-type and mutants structures were known, interaction profiles were generated using those structures. In cases where crystal structures for mutants were absent, generation of local interaction profiles was carried out using known wild-type and modelled mutant structure. Information provided in iATMs could provide a better understanding of correlation between experimental observations and interaction rearrangements due to mutations, leading to better application of derived knowledge towards efficient protein engineering.

## Conclusion

Despite the availability of a large number of structural analysis tools, to the best of our knowledge there is currently no unified platform addressing the rational protein design problem. The web platform iRDP uniquely offers investigators a multi-faceted approach for carrying out rational protein engineering by integrating protein structure and mutation analysis tools. The modules of iRDP server can either be used separately for various independent analyses or as a systematic directed strategy encompassing the steps involved in rational protein engineering. Applications of modules do not limit themselves to the protein stability problem since the information generated comprises of diverse structural features, which can correlate with a wide range of properties in proteins. Investigations carried out using iRDP act as a guide for analysing varied structural features that relate to problems such as pH stability, protein active site analysis, crystallizability, analysis of frames from molecular dynamics simulations and protein structure-function relationships.

The future direction of the iRDP web server aspires towards implementation of sequence-based inputs complementing the existing structure-based input followed by visualization of interaction networks and mutation sites, thereby providing a better structural perspective. We welcome comments and corrections from users to further improve the iRDP server.

## Supporting Information

S1 Fig The workflow of the working modules implemented in the iRDP web server. (TIFF)

S2 Fig The input mutation format for user-defined mutations in the iMutants module of the iRDP web server. (TIF)

S3 Fig Box plot illustrating the % sequence identity among proteins in Thermophilic- Mesophilic (TS-MS) pairs, all TS proteins and all MS proteins. High degree of homology observed between TS-MS protein pairs compared to all-TS and all-MS protein sets.
(TIFF)

S4 Fig The ROC curve for the iStability prediction based on validation dataset of 81 mutations. The neutral-state cut-off values used are labelled near the data points. It is observed that the True-positive rate or sensitivity remains > 0.8 for neutral state cutoff range of 0 (no neutral state) to 2.5. The point shown at origin is just used for joining the lines between data points to the origin.
(TIFF)

S1 File Supplementary references. (PDF)

S1 Table List of protein structure analysis tools. (PDF)

S2 Table List of mutant stability prediction tools. (PDF)

S3 Table List of tools used by iRDP web server for estimation of various structural parameters. (PDF)

S4 Table List of various quantitative parameters (total 288) analyzed by the iCAPS module. (PDF)

S5 Table Details of proteins considered in iCAPS validation. (PDF)

S6 Table Details of proteins used for iStability and iMutants validation. (PDF)

S7 Table Validation of iStability using proline insertion at Ncap of helix and disulfide bond insertion strategy. (PDF)

S8 Table The iMutant analysis on 51 mutations in Arc Repressor protein of bacteriophage P22. Local interaction profile represents number of various interactions and interaction networks of wild-type and mutant residues. The label corresponds to number of IP: ion-pair, IP_Net: ion- pair networks, AP: aromatic aromatic interaction, AP.Net: aromatic aromatic interaction network, AS: aromatic sulphur interactions, AS.Net: aromatic-sulphur interaction network, HB: hydrogen bonds, Disul: disulfide bonds, Cat-pi: cation-pi interactions, Cat-pi_Net: cation-pi interaction networks, Hphob: hydrophobic interactions. The—(hyphen) corresponds to no interaction or interaction networks detected. The Tm value corresponds to the Tm value of Mutant. The results can be accessed following the link http://irdp.ncl.res.in/cgi-bin/result_fetch_MutAna.php?ID=iMutcase.
(PDF)

S9 Table iStability validation analysis showing prediction parameters calculated for different neutral-state energy cut-offs. (PDF)
