# Supplementary figures and images for: Engineering Proteins for Thermostability with iRDP Web Server
Source: PLoS One. 2015 Oct 5;10(10):e0139486. doi: 10.1371/journal.pone.0139486 (PMC4593602; doi:10.1371/journal.pone.0139486)

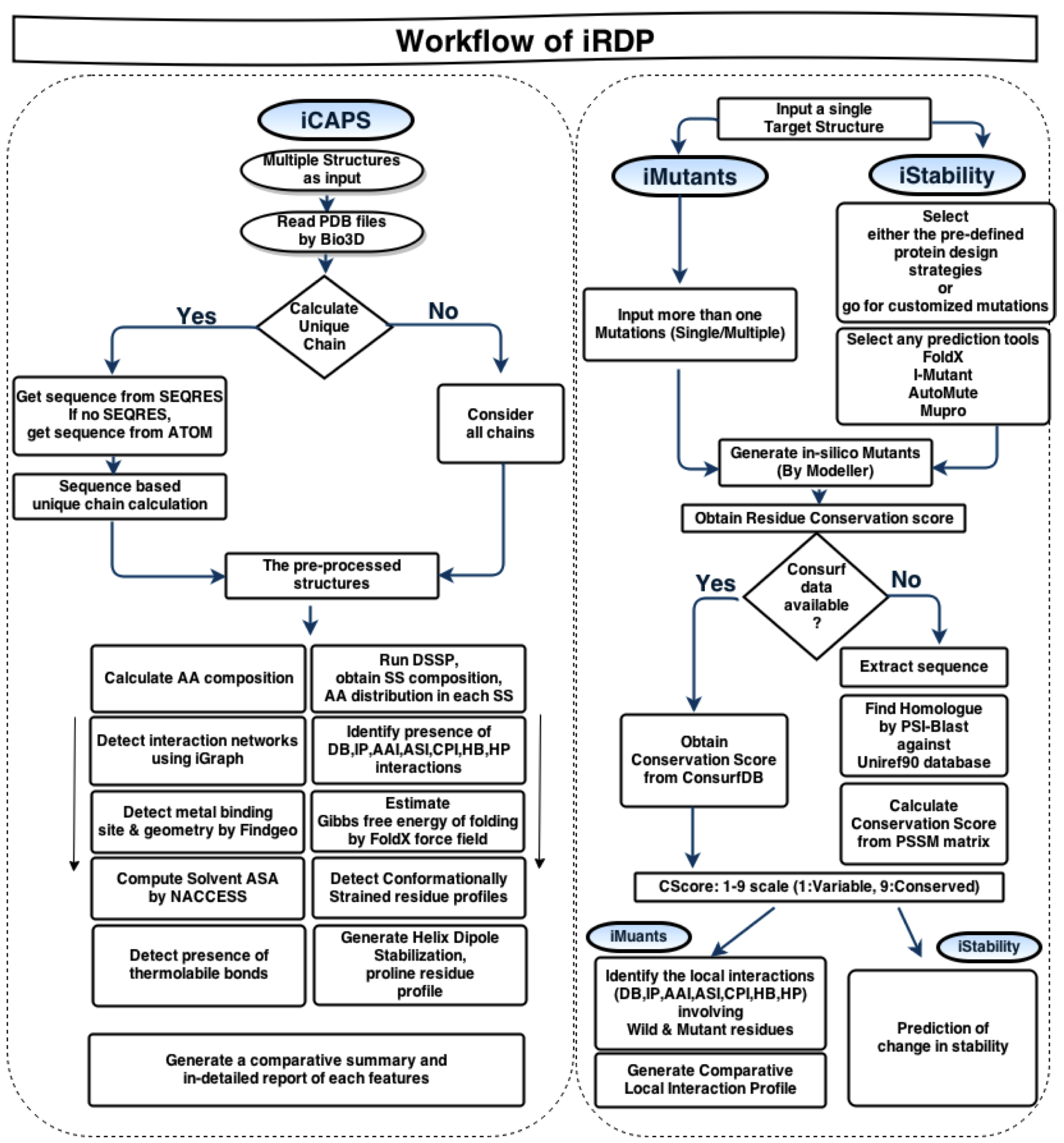

Supplement: S1 Fig — (TIFF) [file pone.0139486.s001.tiff]

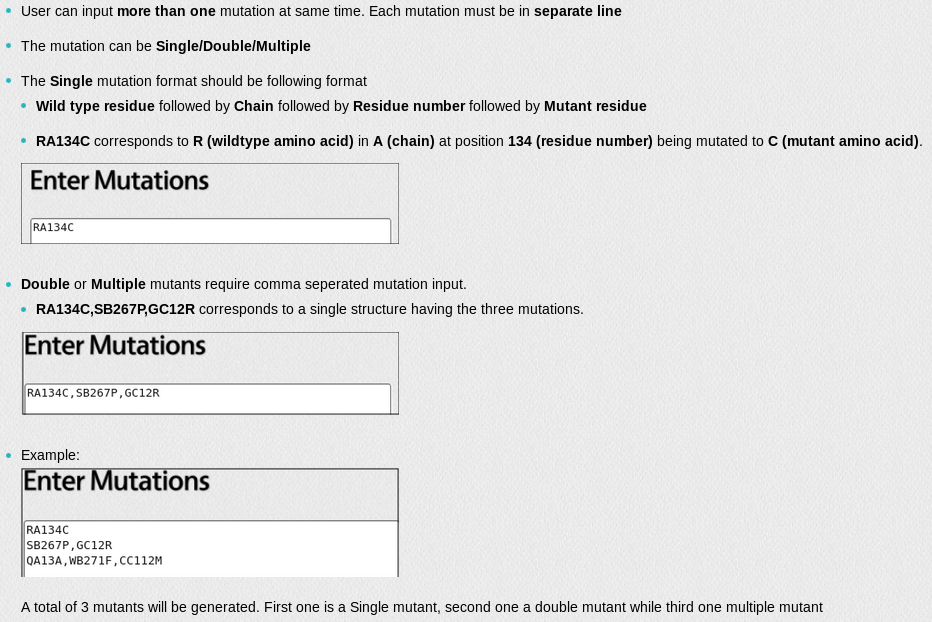

Supplement: S2 Fig — (TIF) [file pone.0139486.s002.tif]

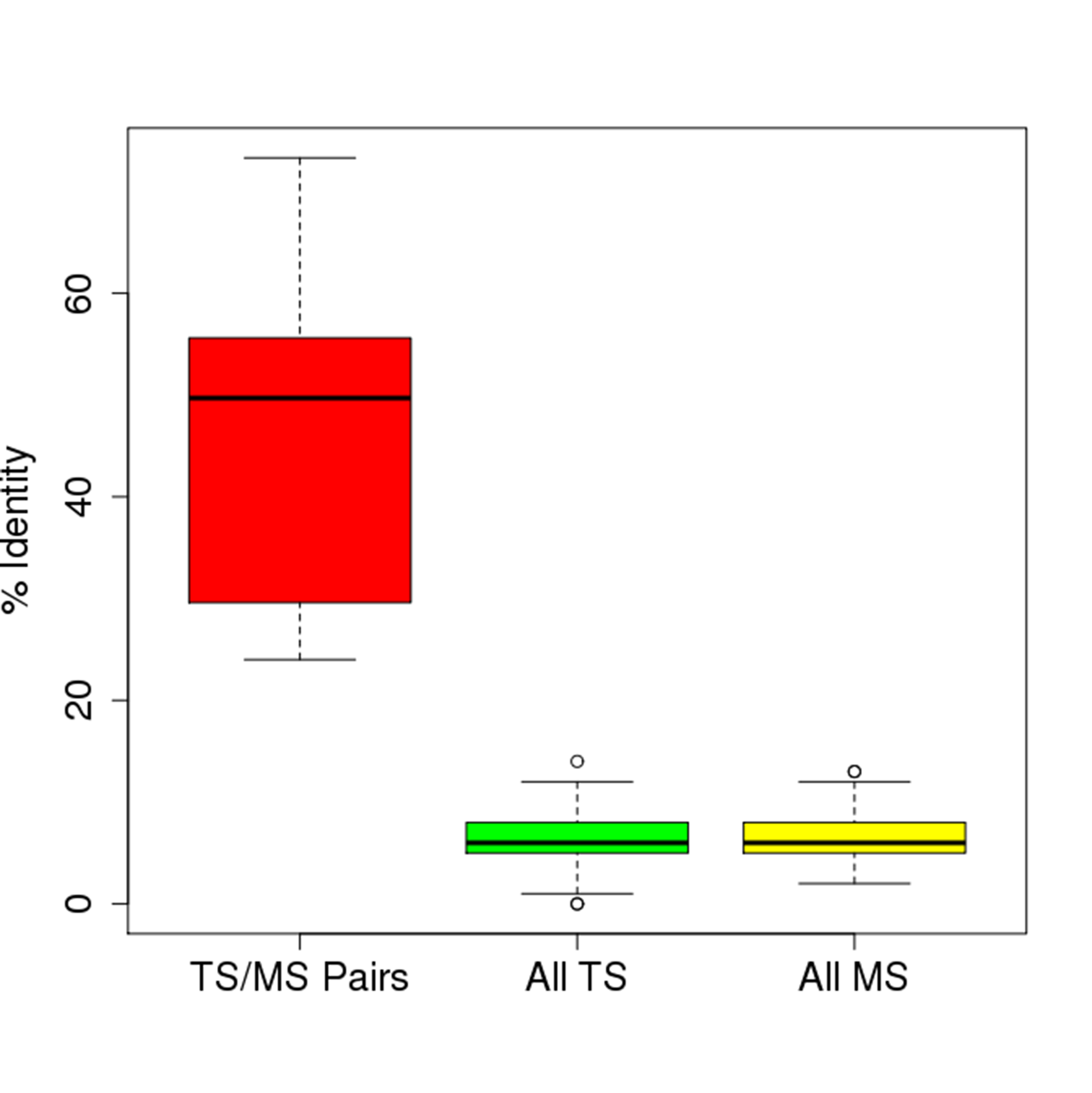

Supplement: S3 Fig — High degree of homology observed between TS-MS protein pairs compared to all-TS and all-MS protein sets. (TIFF) [file pone.0139486.s003.tiff]

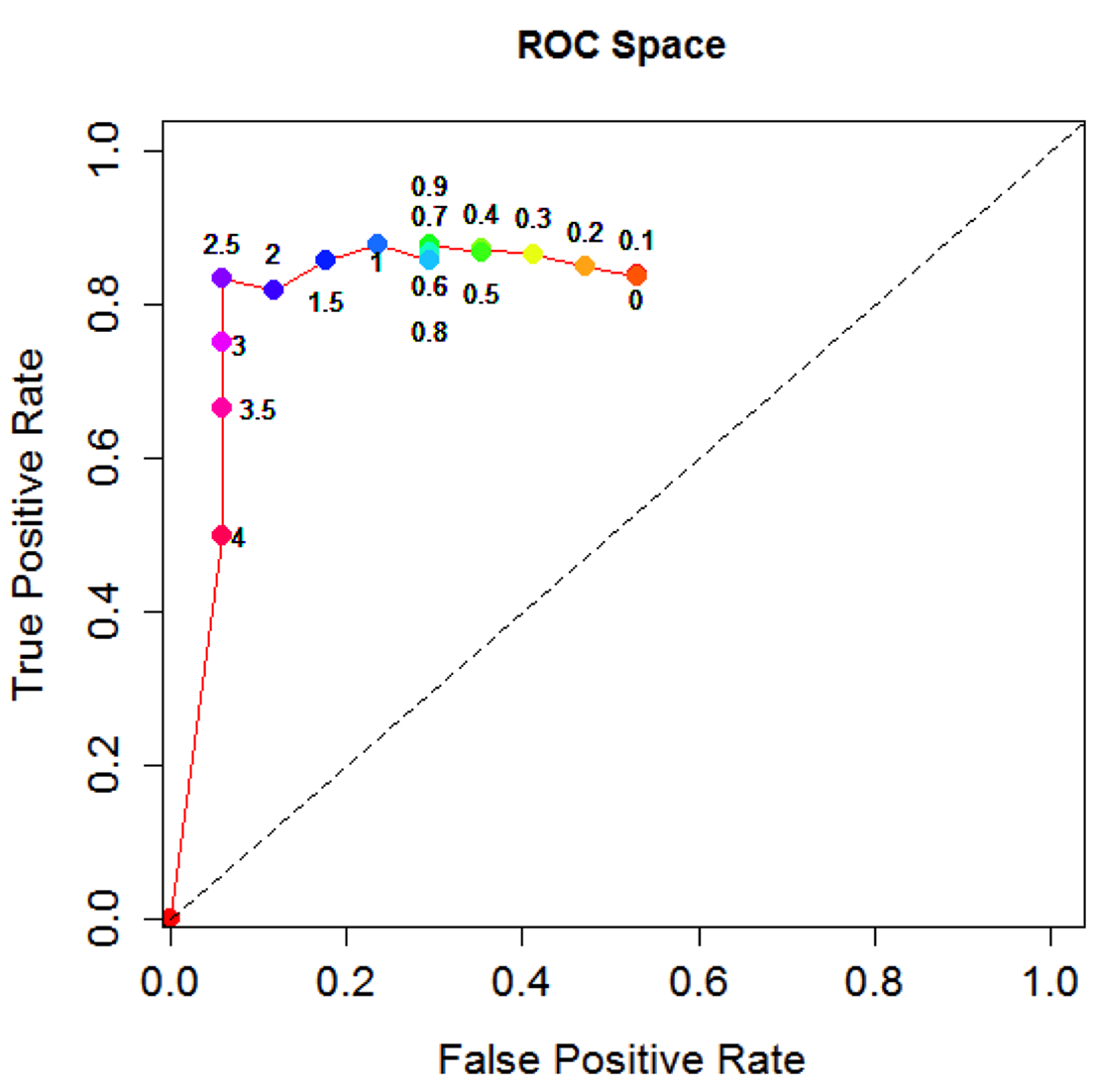

Supplement: S4 Fig — The neutral-state cut-off values used are labelled near the data points. It is observed that the True-positive rate or sensitivity remains > 0.8 for neutral state cutoff range of 0 (no neutral state) to 2.5. The point shown at origin is just used for joining the lines between data points to the origin. (TIFF) [file pone.0139486.s004.tiff]
