# Supplementary material for: Engineering Proteins for Thermostability with iRDP Web Server
Source: PLoS One. 2015 Oct 5;10(10):e0139486. doi: 10.1371/journal.pone.0139486 (PMC4593602; doi:10.1371/journal.pone.0139486)
Supplement: S1 File — (PDF) [file pone.0139486.s005.pdf]

Supplementary References

Andreini C., Cavallaro G. and Lorenzini S. (2012) FindGeo: a tool for determining metal coordination geometry. *Bioinformatics,* **28**, 1658-1660.

Baker E.N. and Hubbard R.E. (1984) Hydrogen bonding in globular proteins. *Progress in biophysics and molecular biology,* **44**, 97-179.

Burley S. and Petsko G. (1985) Aromatic-aromatic interaction: a mechanism of protein structure stabilization. *Science,* **229**, 23-28.

Cabrita L.D., Gilis D., Robertson A.L., Dehouck Y., Rooman M. and Bottomley S.P. (2007) Enhancing the stability and solubility of TEV protease using in silico design. *Protein science,* **16**, 2360-2367.

Capriotti E., Fariselli P. and Casadio R. (2005) I-Mutant2. 0: predicting stability changes upon mutation from the protein sequence or structure. *Nucleic acids research,* **33**, W306-W310.

Cheng J., Randall A. and Baldi P. (2006) Prediction of protein stability changes for single-site mutations using support vector machines. *Proteins: Structure, Function, and Bioinformatics,* **62**, 1125-1132.

Costantini S., Colonna G. and Facchiano A.M. (2008) ESBRI: a web server for evaluating salt bridges in proteins. *Bioinformation,* **3**, 137-138.

Eswar N., Webb B., Marti-Renom M.A., Madhusudhan M., Eramian D., Shen M.y., Pieper U. and Sali A. (2006) Comparative protein structure modeling using Modeller. *Current protocols in bioinformatics*, 5.6. 1-5.6. 30.

Gallivan J.P. and Dougherty D.A. (1999) Cation-π interactions in structural biology. *Proceedings of the National Academy of Sciences,* **96**, 9459-9464.

Giollo M., Martin A.J., Walsh I., Ferrari C. and Tosatto S.C. (2014) NeEMO: a method using residue interaction networks to improve prediction of protein stability upon mutation. *BMC Genomics,* **15**, S7.

Guerois R., Nielsen J.E. and Serrano L. (2002) Predicting changes in the stability of proteins and protein complexes: a study of more than 1000 mutations. *Journal of molecular biology,* **320**, 369-387.

Hazes B. and Dijkstra B.W. (1988) Model building of disulfide bonds in proteins with known three-dimensional structure. *Protein engineering,* **2**, 119-125.

Hubbard S.J. and Thornton J.M. (1993) Naccess. *Computer Program, Department of Biochemistry and Molecular Biology, University College London,* **2**.

Hutchinson E.G. and Thornton J.M. (1996) PROMOTIF—a program to identify and analyze structural motifs in proteins. *Protein Science,* **5**, 212-220.

Kabsch W. and Sander C. (1983) Dictionary of protein secondary structure: pattern recognition of hydrogen-bonded and geometrical features. *Biopolymers,* **22**, 2577-2637.

Kimura S., Kanaya S. and Nakamura H. (1992) Thermostabilization of Escherichia coli ribonuclease HI by replacing left-handed helical Lys95 with Gly or Asn. *Journal of Biological Chemistry,* **267**, 22014-22017.

Laskowski R.A., MacArthur M.W., Moss D.S. and Thornton J.M. (1993) PROCHECK: a program to check the stereochemical quality of protein structures. *Journal of applied crystallography,* **26**, 283-291.

Li C., Heatwole J., Soelaiman S. and Shoham M. (1999) Crystal structure of a thermophilic alcohol dehydrogenase substrate complex suggests determinants of substrate specificity and thermostability. *Proteins,* **37**, 619-627.

Masso M. and Vaisman I.I. (2011), *Engineering in Medicine and Biology Society, EMBC, 2011 Annual International Conference of the IEEE*. IEEE, pp. 3221-3224.

Matsumura M., Signor G. and Matthews B.W. (1989) Substantial increase of protein stability by multiple disulphide bonds. *Nature,* **342**, 291-293.

McDonald I.K. and Thornton J.M. (1994) Satisfying hydrogen bonding potential in proteins. *J Mol Biol,* **238**, 777-793.

Pace C.N. (1992) Contribution of the hydrophobic effect to globular protein stability. *J Mol Biol,* **226**, 29-35.

Parthiban V., Gromiha M.M. and Schomburg D. (2006) CUPSAT: prediction of protein stability upon point mutations. *Nucleic acids research,* **34**, W239-W242.

Reid K.S.C., Lindley P.F. and Thornton J.M. (1985) Sulphur-aromatic interactions in proteins. *FEBS Letters,* **190**, 209-213.

Robinson N.E. (2002) Protein deamidation. *Proceedings of the National Academy of Sciences of the United States of America,* **99**, 5283-5288.

Sathyapriya R. and Vishveshwara S. (2004) Interaction of DNA with clusters of amino acids in proteins. *Nucleic acids research,* **32**, 4109-4118.

Tina K., Bhadra R. and Srinivasan N. (2007) PIC: protein interactions calculator. *Nucleic acids research,* **35**, W473-W476. First published on.

Vieille C. and Zeikus G.J. (2001) Hyperthermophilic enzymes: sources, uses, and molecular mechanisms for thermostability. *Microbiology and molecular biology reviews : MMBR*, **65**, 1-43.

Vriend G. (1990) WHAT IF: a molecular modeling and drug design program. *Journal of molecular graphics*, **8**, 52-56.

Worth C.L., Preissner R. and Blundell T.L. (2011) SDM—a server for predicting effects of mutations on protein stability and malfunction. *Nucleic acids research*, **39**, W215-W222.

Yip K.S., Stillman T.J., Britton K.L., Artymiuk P.J., Baker P.J., Sedelnikova S.E., Engel P.C., Pasquo A., Chiaraluce R., Consalvi V. *et al.* (1995) The structure of Pyrococcus furiosus glutamate dehydrogenase reveals a key role for ion-pair networks in maintaining enzyme stability at extreme temperatures. *Structure (London, England : 1993)*, **3**, 1147-1158.
