# Supplementary material for: Engineering Proteins for Thermostability with iRDP Web Server
Source: PLoS One. 2015 Oct 5;10(10):e0139486. doi: 10.1371/journal.pone.0139486 (PMC4593602; doi:10.1371/journal.pone.0139486)
Supplement: S1 Table — (PDF) [file pone.0139486.s006.pdf]

S1 Table. List of protein structure analysis tools.

Tools	Usefulness	Need for improvements	Module	Reference
**PIC server**	Analysis of various non-bonded interactions at global structural level.	Fail to provide simultaneous analysis of multiple structures. Do not identify interaction networks. Do not consider other structure stabilization factors that are imperative to proficient protein design.	iCAPS	(Tina *et al.*, 2007)
**ESBRI server**				(Costantini *et al.*, 2008)
**Capture**				(Gallivan and Dougherty, 1999)
**What If**				(Vriend, 1990)
