# Supplementary material for: Engineering Proteins for Thermostability with iRDP Web Server
Source: PLoS One. 2015 Oct 5;10(10):e0139486. doi: 10.1371/journal.pone.0139486 (PMC4593602; doi:10.1371/journal.pone.0139486)
Supplement: S2 Table — (PDF) [file pone.0139486.s007.pdf]

S2 Table. List of mutant stability prediction tools.

**Tools**	**Usefulness**	**Need for improvements**	**Module**	**Reference**
**SDM**	Protein stability prediction	Supports the analysis of single mutants. One mutation can be analyzed at single time. Do not estimate evolutionary conservation of the mutation site. Undertake user-specified mutations for analysis while failing to suggest potential mutation sites by implementing known protein-design strategies.	iStability and iMutants	(Worth *et al.*, 2011)
**NeEMO**				(Giollo *et al.*, 2014)
**POPMUSIC**				(Cabrita *et al.*, 2007)
**CUPSAT**				(Parthiban *et al.*, 2006)
