# Supplementary material for: Engineering Proteins for Thermostability with iRDP Web Server
Source: PLoS One. 2015 Oct 5;10(10):e0139486. doi: 10.1371/journal.pone.0139486 (PMC4593602; doi:10.1371/journal.pone.0139486)
Supplement: S3 Table — (PDF) [file pone.0139486.s008.pdf]

S3 Table. List of tools used by iRDP web server for estimation of various structural parameters.

Tools	Purpose	Reference
DSSP	For assignment of secondary structures	(Kabsch and Sander, 1983)
NACCESS	For estimation of residue solvent accessibility	(Hubbard and Thornton, 1993)
Promotif	For detection of β-turns	(Hutchinson and Thornton, 1996)
Procheck	For calculation of conformational parameters	(Laskowski *et al.*, 1993)
HBPLUS	For identification of hydrogen bonds	(McDonald and Thornton, 1994)
SSBOND	For identification of residue pairs for disulfide bond insertion	(Hazes and Dijkstra, 1988)
MODELLER	For generating in-silico mutants	(Eswar *et al.*, 2006)
FindGeo	For analysis of metal binding sites	(Andreini *et al.*, 2012)
FoldX, AUTO-MUTE, I-Mutant 2.0 and Mupro	For prediction of mutant stability	(Capriotti *et al.*, 2005, Cheng *et al.*, 2006, Guerois *et al.*, 2002, Masso and Vaisman, 2011)
