# Supplementary material for: Engineering Proteins for Thermostability with iRDP Web Server
Source: PLoS One. 2015 Oct 5;10(10):e0139486. doi: 10.1371/journal.pone.0139486 (PMC4593602; doi:10.1371/journal.pone.0139486)
Supplement: S4 Table — (PDF) [file pone.0139486.s009.pdf]

S4 Table. List of various quantitative parameters (total 288) analyzed by the iCAPS module.

Property	Quantitative Features	Number of Features	Calculation
**Amino acid (aa) Composition**	Sequence length	1	
	Frequency of 20 Natural amino acids	20	(Vieille and Zeikus, 2001)
	Frequency of Unnatural amino acids	1	
	Frequency of Aromatic residues [FWY]	1	
	Frequency of Uncharged polar residues [NQST]	1	
	Frequency of Positively charged residues [RKH]	1	
	Frequency of Negatively charged residues [DE]	1	
	Ratio of Arg to Lys residue content (Arg/Lys ratio)	1	
**Secondary structure Composition**	Percentage of residues in isolated beta-bridge [B]	1	DSSP is used to calculate secondary structures in proteins. (Kabsch and Sander, 1983)
	Percentage of residues in extended strands [E]	1	
	Percentage of residues in 3-helix (3/10 helix) [G]	1	
	Percentage of residues in pi helix [I]	1	
	Percentage of residues in alpha helix [H]	1	
	Percentage of residues in hydrogen bonded turn [T]	1	
	Percentage of residues in Bend [S]	1	
	Percentage of residues in random coil [C]	1	
**Helix Composition**	Total residues in helix [DSSP notation: H/G/I]	1	
	Frequency of each of the 20 residues in helix.	20	
**Strand Composition**	Total residues in strand [DSSP notation: B/E]	1	
	Frequency of each of the 20 residues in strand.	20	
**Turn Composition**	Total residues in turn [DSSP notation: T]	1	
	Frequency of each of the 20 residues in turn	20	
**Coil Composition**	Total residues in coil [DSSP notation: S/C]	1	
	Frequency of each of the 20 residues in coil	20	
**Ion pairs (IPs)**	Total number of IPs	1	A salt bridge is considered to be formed if the distance between any of the oxygen atoms of acidic residues and the nitrogen atoms of basic residues are within the cut-off distance (default 6 Å).
	Total number of intra-subunit IPs	1	
	Total number of inter-subunit IPs	1	
	Total IPs involving D/E/R/K/H	5	
	Total IPs of type DR/DK/DH/ER/EK/EH	6	
	Total number of buried IPs	1	
	Total number of exposed IPs	1	
	Percentage of isolated IPs, not involved in any network	1	
	Total number of IP-networks	1	
	IP-network details	1	

			(Yip *et al.*, 1995)
**Aromatic pairs (APs)**	Total number of APs<br>Total number of intra-subunit APs<br>Total number of inter-subunit APs<br>Total APs involving F/W/Y<br>Total APs of type FF/FY/FW/YY/YW/WW<br>Total number of buried APs<br>Total number of exposed APs<br>Percentage of isolated APs, not involved in any network<br>Total number of AP-networks<br>AP-network details	1<br>1<br>1<br>3<br>6<br>1<br>1<br>1<br>1<br>1	Aromatic residues interact with each other if the distance between their phenyl ring centroids lies between 4.5 Å- 7.0 Å.<br>A cut-off of dihedral angle between the planes of such interacting aromatic residues can be set between 30° and 90°.<br><br>(Burley and Petsko, 1985)
**Aromatic-sulphor interactions (ASI)**	Total number of ASI<br>Total number of intra-subunit ASI<br>Total number of inter-subunit ASI<br>Total ASI involving F/W/Y/C/M<br>Total ASI of type FC/YC/WC/FM/YM/WM<br>Total number of buried ASI<br>Total number of exposed ASI<br>Percentage of isolated ASI, not involved in any network<br>Total number of ASI-networks<br>ASI-network details	1<br>1<br>1<br>5<br>6<br>1<br>1<br>1<br>1<br>1	Distance between the sulphur atoms of Cys/Met and the aromatic rings of Phe/Tyr/Trp if lie within 5.3 Å (default), they account for aromatic-sulphur interactions.<br><br>(Reid *et al.*, 1985)
**Cation-pi interactions (CPI)**	Total number of CPI<br>Total number of intra-subunit CPI<br>Total number of inter-subunit CPI<br>Total CPI involving KF/KY/KW/RF/RY/RW<br>Total number of buried CPI<br>Total number of exposed CPI<br>Percentage of isolated CPI, not involved in any network<br>Total number of CPI-networks<br>CPI-network details	1<br>1<br>1<br>5<br>1<br>1<br>1<br>1<br>1	A cationic side chain (Lys/Arg) if nearer to an aromatic side chain (Phe/Tyr/Trp) within 6 Å (default) separation, they account for cation-pi interactions.<br><br>(Sathyapriya and Vishveshwara, 2004)
**Disulfide bridges (DB)**	Total number of DB<br>Total number of intra-subunit DB<br>Total number of inter-subunit DB<br>Total number of buried DB<br>Total number of exposed DB<br>Size of loops connecting cys residues [Loop size between 0-10/10-20/20-30/30-40/40-50/>50]<br>Number of DB connecting two periodic (PP) secondary structures (DSSP notation: H/G/I/E)<br>Number of DB connecting two non-periodic (NN) secondary structures (DSSP notation: B/T/C/S)<br>Number of DB connecting periodic and non-periodic secondary structures (NP)	1<br>1<br>1<br>1<br>1<br>6<br>1<br>1<br>1	Pairs of cysteines (sulphur atoms) if fall within 2.2 Å (default) are accounted as disulphide bridges.<br><br>(Matsumura *et al.*, 1989)

**Hydrogen bonds (HB)**	Total number of HB<br>Total number of intra-subunit HB<br>Total number of inter-subunit HB<br>Total number of Mainchain-Mainchain HB (MM)<br>Total number of Mainchain-Sidechain HB (MS or SM)<br>Total number of Sidechain-Sidechain HB (SS)<br>Total number of Charged-Neutral HB (CNHB)<br>Total number of Neutral-Neutral HB (NNHB)	1<br>1<br>1<br>1<br>2<br>1<br>1<br>1	HBPLUS is used to detect the hydrogen bonds.<br><br>(Baker and Hubbard, 1984)
**Hydrophobic interactions (HP)**	Total number of HP<br>Total number of intra-subunit HP<br>Total number of inter-subunit HP<br>Total number of buried HP<br>Total number of exposed HP	1<br>1<br>1<br>1<br>1	The residues ALA, VAL, LEU, ILE, MET, PHE, TRP, PRO and TYR are considered to interact if they fall within 5Å (default) range.<br><br>(Pace, 1992)
**Proline residue profile**	Total number of proline residue<br>Frequency of proline residues in helices/strands/turns/coils<br>Total number of buried proline residues<br>Total number of exposed proline residues<br>Total number of prolines present in beta-turns<br>Total number of prolines, present at 2nd-position of beta-turns<br>Total number of prolines, at N-cap position of helices.	1<br>4<br>1<br>1<br>1<br>1<br>1	(Li *et al.*, 1999)<br><br>(Suzuki *et al.*, 1987)
**Analysis of Solvent Accessible Surface Area (ASA)**	ASA of all-atoms.<br>ASA of all side-chains atoms.<br>ASA of all main-chain atoms.<br>ASA of non-polar side-chain atoms (NP)<br>ASA of polar side-chain atoms (P)<br>Ratio of Non-polar to polar ASA of side-chain atoms (NP/P ratio)<br>ASA of all C/N/O/S atoms	1<br>1<br>1<br>1<br>1<br>1<br>4	NACCESS is used to calculate the ASA values.<br><br>(Hubbard and Thornton, 1993)
**Thermolabile bond profile**	Total number of thermolabile (TL) bonds.<br>Number of TL bonds of type NG/NA/NS/QG/QA/QS<br>Number of TL bonds where the nucleophilic attack distance is < 4Å	1<br>6<br>1	(Robinson, 2002)
**Helix dipole stabilization profile**	Total number of Helices<br>Number of dipole-stabilized helices.<br>Number of helices stabilized at their N/C/NC terminal.<br>Number of helices whose dipoles are stabilized at N-2/N-1/N/N+1/N+2 positions.<br>Number of helices whose dipoles are stabilized at C-2/C-1/C/C+1/C+2 positions	1<br>1<br>3<br>5<br>5	Helices are identified using DSSP. Identified helices are checked for presence of charged residues at their N- and C-terminals.

			(Vieille and Zeikus, 2001)
**Conformationally strained residue profile**	Total number of conformationally strained (CS) residues.<br>Number of CS residues in L/l/~l region of Ramachandran plot<br>Details of the CS residues	1<br>3<br>1	Procheck is used to identify conformationally strained residues.<br>(Kimura *et al.*, 1992)
**Metal binding summary**	Total number of metals.<br>Details of metal binding sites.	1<br>1	Findgeo (Andreini, Cavallaro and Lorenzini, 2012) program is used to identify metal binding sites.
**Gibbs energy of folding**	Gibbs energy of folding decomposed into individual energies	23	(Guerois, Nielsen and Serrano, 2002)
