# Supplementary material for: Engineering Proteins for Thermostability with iRDP Web Server
Source: PLoS One. 2015 Oct 5;10(10):e0139486. doi: 10.1371/journal.pone.0139486 (PMC4593602; doi:10.1371/journal.pone.0139486)
Supplement: S5 Table — (PDF) [file pone.0139486.s010.pdf]

**S5 Table. Details of proteins considered in iCAPS validation.**

| Protein family | TS | | | | | | | | MS | | | | | | | | R.M.S.D (in Å) | Seq. ID (in %) |
|---|---|---|---|---|---|---|---|---|---|---|---|---|---|---|---|---|---|---|
| | Source | TL (in °C) | PDB entry | Seq. Length | Resolution (Å) | SCOP Class | Mol. Wt. (in Da) | Oligomeric state | Source | TL (in °C) | PDB entry | Seq. Length | Resolution (Å) | SCOP Class | Mol. Wt. (in Da) | Oligomeric state | | |
| **Citrate synthase** | *Pyrococcus furiosus* | 100 | **1AJ8** | 371 | 1.9 | All alpha proteins | 42340.4 | Dimer | *Gallus gallus* | 37 | **1CSH** | 435 | 1.6 | All alpha proteins | 48175.4 | Dimer | 1.68 | 26.2 |
| **Malate dehydrogenase** | *Thermus flavus* | 70–75 | **1BDM** | 327 | 2.5 | Alpha and beta proteins | 35465 | Dimer | *Sus scrofa* | 37 | **4MDH** | 334 | 2.5 | Alpha and beta proteins | 36394.3 | Dimer | 0.94 | 54.1 |
| **Rubredoxin** | *Pyrococcus furiosus* | 100 | **1CAA** | 53 | 108 | Small proteins | 5900.58 | Monomer | *Desulfovibrio vulgaris* | 34–37 | **8RXN** | 52 | 1 | Small proteins | 5578.21 | Monomer | 0.69 | 66.7 |
| **Cyclodextrin** | *Thermoanaerobacterium thermosulfurigenes* | 60 | **1CIU** | 683 | 2.3 | All beta proteins | 75498.8 | Monomer | *Bacillus circulans* | 30–40 | **1CDG** | 686 | 2 | All beta proteins | 74576.2 | Monomer | 0.7 | 70.5 |
| **Glutamate dehydrogenase** | *Pyrococcus furiosus* | 75–100 | **1GTM** | 419 | 2.2 | Alpha and beta proteins | 46983.1 | Hexamer | *Clostridium symbiosum* | 30–37 | **1HRD** | 449 | 1.96 | Alpha and beta proteins | 49216.1 | Hexamer | 1.38 | 34.3 |
| **Lactate dehydrogenase** | *Bacillus stearothermophilus* | 40–65 | **1LDN** | 316 | 2.5 | Alpha and beta proteins | 34745.8 | Tetramer | *Plasmodium falciparum* | 37 | **1LDG** | 316 | 1.74 | Alpha and beta proteins | 34163 | Tetramer | 1.25 | 28.4 |
| **Thermolysin and neutral** | *Bacillus thermoproteolyticus* | 52.5 | **1LNF** | 316 | 1.7 | Alpha and beta proteins | 34362.6 | Monomer | *Bacillus cereus* | 30 | **1NPC** | 317 | 2 | Alpha and beta proteins | 33816.8 | Monomer | 0.86 | 73.3 |
| **3-Phosphoglycerate kinase** | *Bacillus stearothermophilus* | 40–65 | **1PHP** | 394 | 1.65 | Alpha and beta proteins | 42790.5 | Monomer | *Saccharomyces cerevisiae* | 25–30 | **1QPG** | 415 | 2.4 | Alpha and beta proteins | 44641.6 | Monomer | 1.28 | 51.4 |
| **CheY** | *Thermotoga maritima* | 90 | **1TMY** | 120 | 1.9 | Alpha and beta proteins | 13234.8 | Monomer | *Escherichia coli* | 37 | **3CHY** | 128 | 1.66 | Alpha and beta proteins | 13981.2 | Monomer | 1.39 | 28.6 |
| **Methionine aminopeptidase** | *Pyrococcus furiosus* | 100 | **1XGS** | 295 | 1.75 | Alpha and beta proteins | 32888.7 | Dimer | *Escherichia coli* | 37 | **1MAT** | 264 | 2.4 | Alpha and beta proteins | 29371 | Monomer | 1.39 | 30.6 |
| **Endo-1,4-b Xylanase** | *Thermomyces lanuginosus* | 50 | **1YNA** | 194 | 1.55 | All beta proteins | 21312 | Monomer | *Bacillus circulans* | 30–40 | **1XNB** | 185 | 1.49 | All beta proteins | 20409.2 | Monomer | 1.14 | 50.9 |
| **Adenylate kinase** | *Bacillus stearothermophilus* | 40–65 | **1ZIN** | 217 | 1.65 | Alpha and beta proteins | 24175 | Monomer | *Sacchromyces cerevisae* | 25–30 | **1AKY** | 220 | 1.63 | Alpha and beta proteins | 24068.7 | Monomer | 1.22 | 42 |

| | | | | | | | | | | | | | | | | |
|---|---|---|---|---|---|---|---|---|---|---|---|---|---|---|---|---|
| **Ferredoxin** | *Bacillus thermoproteolyticus* | 52.5 | **1IQZ** | 81 | 2.3 | Alpha and beta proteins | 8773.65 | Monomer | *Clostridium acidurici* | 19–37 | **1FCA** | 55 | 1.8 | Alpha and beta proteins | 5496.18 | Monomer | 1.27 | 24 |
| **Inorganic pyrophosphatase** | *Thermus thermophilus* | 70–75 | **2PRD** | 174 | 2 | All beta proteins | 19110 | Hexamer | *Escherichia coli* | 37 | **1INO** | 175 | 2.2 | All beta proteins | 19597.5 | Hexamer | 1.1 | 48.5 |
| **Manganese superoxide dismutase** | *Thermus thermophilus* | 70–75 | **3MDS** | 203 | 1.8 | All alpha proteins | 23129.5 | Tetramer | *Homo sapiens* | 37 | **1QNM** | 198 | 2.3 | All alpha proteins | 22219.3 | Tetramer | 1.17 | 53.2 |
| **Phosphofructokinase** | *Bacillus stearothermophilus* | 40–65 | **3PFK** | 319 | 2.4 | Alpha and beta proteins | 34167.1 | Tetramer | *Escherichia coli* | 37 | **2PFK** | 320 | 2.4 | Alpha and beta proteins | 34885.3 | Tetramer | 0.87 | 57.1 |
