# Supplementary material for: Engineering Proteins for Thermostability with iRDP Web Server
Source: PLoS One. 2015 Oct 5;10(10):e0139486. doi: 10.1371/journal.pone.0139486 (PMC4593602; doi:10.1371/journal.pone.0139486)
Supplement: S6 Table — (PDF) [file pone.0139486.s011.pdf]

.

S6 Table. Details of proteins used for iStability and iMutants validation.

PDB ID	Resolution	Macromolecule Name	Source	Seq. Length	Mol. Wt. (in Da)	Oligomeric state	SCOP Class	PubMed ID
1A5E	-	TUMOR SUPPRESSOR P16INK4A	*Homo sapiens*	156	16554.8	Monomer	Alpha and beta proteins	9660926
1BCX	1.81	XYLANASE	*Bacillus circulans*	185	20383.2	Monomer	All beta proteins	8019418
1BNI	2.1	BARNASE	*Bacillus amyloliquefaciens*	110	12398.8	Trimer	Alpha and beta proteins	8254677
1CAH	1.88	CARBONIC ANHYDRASE II	*Homo sapiens*	259	29158.1	Monomer	All beta proteins	1474587
1CSP	2.45	COLD SHOCK PROTEIN B(CSPB)	*Bacillus subtilis*	67	7372.21	Monomer	All beta proteins	8321288
1EY0	1.6	STAPHYLOCOCCAL NUCLEASE	*Staphylococcus aureus*	149	16843.5	Monomer	All beta proteins	11023780
1EYA	2	STAPHYLOCOCCAL NUCLEASE	*Staphylococcus aureus*	149	16772.5	Monomer	All beta proteins	11023780
1FYH	2.04	INTERFERON-GAMMA	*Homo sapiens*	258	30146.5	Dimer	All alpha proteins	11250200
1HTI	2.8	TRIOSEPHOSPHATE ISOMERASE	*Homo sapiens*	248	26570.5	Dimer	Alpha and beta proteins	8061610
1KEV	2.05	NADP-DEPENDENT ALCOHOL DEHYDROGENASE	*Clostridium beijerinckii*	351	37763.2	Tetramer	All beta proteins	15299659
1LTA	2.2	HEAT-LABILE ENTEROTOXIN, SUBUNIT A	*Escherichia coli*	188	21641	Tetramer	All beta proteins	7997185
1LVE	1.95	LEN, A VARIABLE DOMAIN FROM KAPPA-4 TYPE	*Homo sapiens*	122	13423	Monomer	All beta proteins	9683271
1LZ1	1.5	HUMAN LYSOZYME	*Homo sapiens*	130	14720.8	Monomer	Alpha and beta proteins	7334520
1MGR	1.7	Guanyl-specific ribonuclease Sa3	*Streptomyces aureofaciens*	99	11057.2	Monomer	Alpha and beta proteins	12228255
1PGA	2.07	PROTEIN G	*Streptococcus sp. GX7805*	56	6201.84	Monomer	Alpha and beta proteins	8161530
1PII	2	N-(5'PHOSPHORIBOSYL)ANTHRANILATE ISOMERASE	*Escherichia coli*	452	49413.4	Monomer	Alpha and beta proteins	1738159
1PIN	1.35	PEPTIDYL-PROLYL CIS-TRANS ISOMERASE	*Homo sapiens*	163	18271.4	Monomer	All beta proteins	9200606
1PLC	1.33	PLASTOCYANIN	*Populus nigra*	99	10493.7	Monomer	All beta proteins	1492962
1PYL	1.51	ribonuclease	*Streptomyces aureofaciens*	97	10909.1	Dimer	Alpha and beta proteins	15213380
1RGG	1.2	RIBONUCLEASE	*Streptomyces aureofaciens*	96	10582.6	Dimer	Alpha and beta proteins	15299705
1ROP	1.7	ROP PROTEIN	*Escherichia coli*	63	7237.09	Monomer	All alpha proteins	3681971
1RTP	2	ALPHA-PARVALBUMIN	*Rattus rattus*	109	11813.4	Trimer	All alpha proteins	8289291
1SBT	2.5	SUBTILISIN BPN'	*Bacillus amyloliquefaciens*	275	27552.7	Monomer	Alpha and beta proteins	5160720
1STN	1.7	STAPHYLOCOCCAL NUCLEASE	*Staphylococcus aureus*	149	16843.5	Monomer	All beta proteins	1896431
1SUE	1.8	SUBTILISIN BPN'	*Bacillus amyloliquefaciens*	266	26649.7	Monomer	Alpha and beta proteins	-
1UOK	2	OLIGO-1,6-GLUCOSIDASE	*Bacillus cereus*	558	66090.8	Monomer	All beta proteins	9193006
1WE4	1.7	Beta-lactamase Toho-1	*Escherichia coli*	262	28368.4	Monomer	Alpha and beta proteins	15595829
1Z7X	1.95	Ribonuclease inhibitor	*Homo sapiens*	461	50018.7	Monomer	Alpha and beta proteins	17350650
1ZW7	-	Ubiquitin	*Saccharomyces cerevisiae*	82	9177.45	Monomer	Alpha and beta proteins	16713063
2A36	-	Protein E(sev)2B	*Drosophila melanogaster*	59	6868.68	Monomer	All beta proteins	16300404
2AFG	2	ACIDIC FIBROBLAST GROWTH FACTOR	*Homo sapiens*	140	15858	Tetramer	All beta proteins	8652550
2IMM	2	IGA-KAPPA MCPC603 FV (LIGHT CHAIN)	*Mus musculus*	114	12341.8	Monomer	All beta proteins	1602480
2LZM	1.7	T4 LYSOZYME	*Enterobacteria phage T4*	164	18662.6	Monomer	Alpha and beta proteins	3586019
2RN2	1.48	RIBONUCLEASE H	*Escherichia coli*	155	17623.1	Monomer	Alpha and beta proteins	1311386
3CI2	-	CHYMOTRYPSIN INHIBITOR 2	*Hordeum vulgare*	66	7547.91	Monomer	Alpha and beta proteins	1748996
3GLY	2.2	GLUCOAMYLASE-471	*Aspergillus awamori*	470	50451.3	Monomer	All alpha proteins	8176747
3MBP	1.7	MALTODEXTRIN-BINDING PROTEIN	*Escherichia coli*	370	40753.6	Monomer	Alpha and beta proteins	9309217
4DFR	1.7	DIHYDROFOLATE REDUCTASE	*Escherichia coli*	159	18020.5	Dimer	Alpha and beta proteins	6815178

5AZU	1.9	AZURIN	*Pseudomonas aeruginosa*	128	13961.9	Tetramer	All beta proteins	1942029
9RNT	1.5	RIBONUCLEASE T1	*Aspergillus oryzae*	104	11094.8	Monomer	Alpha and beta proteins	1960730

The - in Resolution column indicates absence of Resolution information, since the structures are solved by NMR.
