# Supplementary material for: Engineering Proteins for Thermostability with iRDP Web Server
Source: PLoS One. 2015 Oct 5;10(10):e0139486. doi: 10.1371/journal.pone.0139486 (PMC4593602; doi:10.1371/journal.pone.0139486)
Supplement: S7 Table — (PDF) [file pone.0139486.s012.pdf]

S7 Table. Validation of iStability using proline insertion at Ncap of helix and disulfide bond insertion strategy.

PDB ID	Protein	Organisms	Mutation	Experiment*	iStability **	FoldX energy (kcal/mol)**	PUBMED Id
**Stabilization by insertion of Proline residues at N-cap of Helices**							
1HTI	Triosephosphate isomerase	*Homo sapiens*	A215P	I	I	-0.04	8672446
1LZ1	Lysozyme	*Homo sapiens*	V110P	I	I	-2.11	1911779
1RTP	Alpha-parvalbumin	*Rattus rattus*	H26P	I (5.6^{o}C)	I	-2.11	12974622
1UOK	oligo-1, 6-glucosidase	*Bacillus cereus*	N109P	I	I	-2.01	8001545
			E175P	I	I	-0.19	
			T261P	I	I	-2.53	
			E270P	I	I	-1.59	
			I403P	I	I	-0.86	
1KEV	Alcohol dehydrogenase	*Clostridium beijerinckii*	A177P	I (0.5^{o}C)	I	-1.57	9836874
			L316P	I (10.8^{o}C)	D	2.2	
2LZM	Bacteriophage T4 lysozyme	*Enterobacteria phage T4*	K60P	I (0.3^{o}C)	I	-0.69	1457724
**Stabilization by insertion of Disulfide bridges**							
1BNI	Barnase	*Bacillus amyloliquefaciens*	A43C,S80C	I	D	0.91	8476861
			T70C,S92C	D	D	1.06	
5AZU	Azurin	*Pseudomonas aeruginosa*	D62C,K74C	I	D	0.76	15449946
1BCX	Xylanase	*Bacillus circulans*	V98C,A152C	I	I	-0.49	17141401
			S100C,N148C	I	D	0.75	
1CAH	Carbonic anhydrase II	*Homo sapiens*	L60C,S173C	I	I	-3.29	10794421
			A38C,A258C	D	D	1.15	
			S99C,V242C	I	D	1.52	
1PLC	Plastocyanin	*Populus nigra*	I21C,E25C	I	D	3.38	11679761
1WE4	b-lactamase	*Escherichia coli*	C69C,G238C	I	D	0.97	15595829
1LTA	Cholera toxin	*Vibrio cholerae*	N40C,G166C	I	D	9.07	9416616
3CI2	Chymotrypsin inhibitor-2	*Homo sapiens*	T22C,V82C	I	I	-4.48	11045611
4DFR	Dihydrofolatereductase	*Escherichia coli*	P39C,C85C	I	D	3.14	3304420
3GLY	Glucoamylase	*Aspergillus awamori*	T72C,A471C	N	I	-0.11	9749918
			T246C,C320C	I	D	1.03	8679632
1PII	Indoleglycerol-phosphate synthase	*Escherichia coli*	T3C,R189C	I	D	3.46	11856350
1FYH	Interferon-gamma	*Homo sapiens*	E7C,S69C	I	D	0.76	8931130
1EYA	Nuclease V8	*Staphylococcus aureus*	Q80C,K116C	I	D	0.58	8756688
			N118C,D77C	D	D	3.66	
1EY0	Nuclease V8		N118C,G79C	I	I	-0.34	
1Z7X	Ribonuclease I	*Homo sapiens*	A4C,V118C	I	D	3.47	10920260
1SBT	Subtilisin BPN	*Bacillus*	A26C,A232C	D	D	2.69	2504281

		amyloliquefaciens					
			D41C,G80C	D	I	-6.25	
			T22C,S87C	I	I	-0.69	3476160
1SUE			A29C,M119C	D	D	3.88	
			D36C,P210C	D	I	-0.31	2504281
			V148C,N243C	D	D	3.51	
			A22C,A87C	I	I	-0.92	3476160

* The labels I, D and N correspond to an increase, decrease and no change in stability respectively for the mutations as inferred from experiment. The values with unit kcal/mol represent ddG value (Change in free energy of unfolding, Mutant-Wild-type) while values those with unit ˚C represent dTm value (Change in midpoint temperature of the thermal unfolding, Mutant-Wild-type) as inferred from the experiment. A positive value represents an increase in stability. ** Two states of iStability predictions considered are: I (FoldX energy < 0) representing increased stability and D (FoldX energy > 0) representing decreased stability.
