# Supplementary material for: Engineering Proteins for Thermostability with iRDP Web Server
Source: PLoS One. 2015 Oct 5;10(10):e0139486. doi: 10.1371/journal.pone.0139486 (PMC4593602; doi:10.1371/journal.pone.0139486)
Supplement: S8 Table — Local interaction profile represents number of various interactions and interaction networks of wild-type and mutant residues. The label corresponds to number of IP: ion-pair, IP_Net: ion- pair networks, AP: aromatic aromatic interaction, AP.Net: aromatic aromatic interaction network, AS: aromatic sulphur interactions, AS.Net: aromatic-sulphur interaction network, HB: hydrogen bonds, Disul: disulfide bonds, Cat-pi: cation-pi interactions, Cat-pi_Net: cation-pi interaction networks, Hphob: hydrophobic interactions. The—(hyphen) corresponds to no interaction or interaction networks detected. The Tm value corresponds to the Tm value of Mutant. The results can be accessed following the link http://irdp.ncl.res.in/cgi-bin/result_fetch_MutAna.php?ID=iMutcase. (PDF) [file pone.0139486.s013.pdf]

S8 Table. The iMutant analysis on 51 mutations in Arc Repressor protein of bacteriophage P22. Local interaction profile represents number of various interactions and interaction networks of wild-type and mutant residues. The label corresponds to number of IP: ion-pair, IP_Net: ion-pair networks, AP: aromatic aromatic interaction, AP.Net: aromatic aromatic interaction network, AS: aromatic sulphur interactions, AS.Net: aromatic-sulphur interaction network, HB: hydrogen bonds, Disul: disulfide bonds, Cat-pi: cation-pi interactions, Cat-pi_Net: cation-pi interaction networks, Hphob: hydrophobic interactions. The - (hyphen) corresponds to no interaction or interaction networks detected. The Tm value corresponds to the Tm value of Mutant. The results can be accessed following the link http://irdp.ncl.res.in/cgi-bin/result_fetch_MutAna.php?ID=iMutcase.

No	Type	Chain	ResNo	ResID	Local Interaction Profile											T_m (obs) °C
					IP	IP_Net	AP	AP.Net	AS	AS.Net	HB	Disul	Cat-pi	Cat-pi_Net	Hphob	
**Highly Unstable Mutations**																
1	wild	A	22	V	-	-	-	-	-	-	2	-	-	-	7	<20
	mut	A	22	A	-	-	-	-	-	-	2	-	-	-	2	
2	wild	A	36	E	2	1	-	-	-	-	2	-	-	-	-	<20
	mut	A	36	A	-	-	-	-	-	-	1	-	-	-	2	
3	wild	A	37	I	-	-	-	-	-	-	3	-	-	-	9	<20
	mut	A	37	A	-	-	-	-	-	-	2	-	-	-	3	
4	wild	A	41	V	-	-	-	-	-	-	2	-	-	-	6	<20
	mut	A	41	A	-	-	-	-	-	-	1	-	-	-	3	
5	wild	A	45	F	-	-	-	-	-	-	-	-	-	-	6	<20
	mut	A	45	A	-	-	-	-	-	-	-	-	-	-	2	
**Unstable Mutations**																
6	wild	A	10	F	-	-	1	1	-	-	2	-	-	-	6	40.6
	mut	A	10	A	-	-	-	-	-	-	2	-	-	-	-	
7	wild	A	12	L	-	-	-	-	-	-	2	-	-	-	6	42.3
	mut	A	12	A	-	-	-	-	-	-	2	-	-	-	2	
8	wild	A	14	W	-	-	2	1	-	-	2	-	-	-	1-	31.5
	mut	A	14	A	-	-	-	-	-	-	1	-	-	-	5	
9	wild	A	15	P	-	-	-	-	-	-	2	-	-	-	4	46.6
	mut	A	15	A	-	-	-	-	-	-	2	-	-	-	2	
10	wild	A	19	L	-	-	-	-	-	-	2	-	-	-	4	48.3
	mut	A	19	A	-	-	-	-	-	-	2	-	-	-	-	
11	wild	A	21	L	-	-	-	-	-	-	3	-	-	-	2	39.6
	mut	A	21	A	-	-	-	-	-	-	2	-	-	-	1	
12	wild	A	29	N	-	-	-	-	-	-	2	-	-	-	-	45.3
	mut	A	29	A	-	-	-	-	-	-	1	-	-	-	-	
13	wild	A	30	G	-	-	-	-	-	-	2	-	-	-	-	47.9
	mut	A	30	A	-	-	-	-	-	-	2	-	-	-	-	
14	wild	A	31	R	1	1	-	-	-	-	1	-	-	-	-	37.1
	mut	A	31	A	-	-	-	-	-	-	1	-	-	-	-	
15	wild	A	32	S	-	-	-	-	-	-	1	-	-	-	-	33.5
	mut	A	32	A	-	-	-	-	-	-	1	-	-	-	-	
16	wild	A	33	V	-	-	-	-	-	-	2	-	-	-	3	44.1
	mut	A	33	A	-	-	-	-	-	-	1	-	-	-	3	
17	wild	A	38	Y	-	-	1	1	1	-	3	-	-	-	4	33
	mut	A	38	A	-	-	-	-	-	-	2	-	-	-	1	
18	wild	A	40	R	2	1	-	-	-	-	3	-	-	-	-	31.2
	mut	A	40	A	-	-	-	-	-	-	1	-	-	-	2	

**19**	**wild**	**A**	**42**	**M**	**-**	**-**	**-**	**-**	**1**	**-**	**1**	**-**	**-**	**-**	**3**	**35.6**
	**mut**	**A**	**42**	**A**	**-**	**-**	**-**	**-**	**-**	**-**	**1**	**-**	**-**	**-**	**1**	
**20**	**wild**	**A**	**44**	**S**	**-**	**-**	**-**	**-**	**-**	**-**	**1**	**-**	**-**	**-**	**-**	**46.3**
	**mut**	**A**	**44**	**A**	**-**	**-**	**-**	**-**	**-**	**-**	**2**	**-**	**-**	**-**	**2**	
**21**	**wild**	**A**	**47**	**K**	**-**	**-**	**-**	**-**	**-**	**-**	**3**	**-**	**-**	**-**	**-**	**47.2**
	**mut**	**A**	**47**	**A**	**-**	**-**	**-**	**-**	**-**	**-**	**1**	**-**	**-**	**-**	**-**	
**22**	**wild**	**A**	**48**	**E**	**-**	**-**	**-**	**-**	**-**	**-**	**1**	**-**	**-**	**-**	**-**	**43.2**
	**mut**	**A**	**48**	**A**	**-**	**-**	**-**	**-**	**-**	**-**	**1**	**-**	**-**	**-**	**1**	
**23**	**wild**	**A**	**49**	**G**	**-**	**-**	**-**	**-**	**-**	**-**	**-**	**-**	**-**	**-**	**-**	**48.7**
	**mut**	**A**	**49**	**A**	**-**	**-**	**-**	**-**	**-**	**-**	**-**	**-**	**-**	**-**	**-**	
**24**	**wild**	**A**	**50**	**R**	**1**	**-**	**-**	**-**	**-**	**-**	**3**	**-**	**-**	**-**	**-**	**47.9**
	**mut**	**A**	**50**	**A**	**-**	**-**	**-**	**-**	**-**	**-**	**3**	**-**	**-**	**-**	**1**	
**25**	**wild**	**A**	**51**	**I**	**-**	**-**	**-**	**-**	**-**	**-**	**1**	**-**	**-**	**-**	**2**	**50.9**
	**mut**	**A**	**51**	**A**	**-**	**-**	**-**	**-**	**-**	**-**	**1**	**-**	**-**	**-**	**2**	
**Mutations having Near Wild-type Stability**																
**26**	**wild**	**A**	**1**	**M**	**-**	**-**	**-**	**-**	**-**	**-**	**2**	**-**	**-**	**-**	**-**	**58**
	**mut**	**A**	**1**	**A**	**-**	**-**	**-**	**-**	**-**	**-**	**-**	**-**	**-**	**-**	**1**	
**27**	**wild**	**A**	**2**	**K**	**-**	**-**	**-**	**-**	**-**	**-**	**-**	**-**	**-**	**-**	**-**	**58.7**
	**mut**	**A**	**2**	**A**	**-**	**-**	**-**	**-**	**-**	**-**	**-**	**-**	**-**	**-**	**1**	
**28**	**wild**	**A**	**3**	**G**	**-**	**-**	**-**	**-**	**-**	**-**	**-**	**-**	**-**	**-**	**-**	**58.1**
	**mut**	**A**	**3**	**A**	**-**	**-**	**-**	**-**	**-**	**-**	**-**	**-**	**-**	**-**	**1**	
**29**	**wild**	**A**	**4**	**M**	**-**	**-**	**-**	**-**	**-**	**-**	**2**	**-**	**-**	**-**	**-**	**59.2**
	**mut**	**A**	**4**	**A**	**-**	**-**	**-**	**-**	**-**	**-**	**1**	**-**	**-**	**-**	**-**	
**30**	**wild**	**A**	**5**	**S**	**-**	**-**	**-**	**-**	**-**	**-**	**2**	**-**	**-**	**-**	**-**	**57.5**
	**mut**	**A**	**5**	**A**	**-**	**-**	**-**	**-**	**-**	**-**	**-**	**-**	**-**	**-**	**-**	
**31**	**wild**	**A**	**6**	**K**	**1**	**1**	**-**	**-**	**-**	**-**	**2**	**-**	**-**	**-**	**-**	**59.6**
	**mut**	**A**	**6**	**A**	**-**	**-**	**-**	**-**	**-**	**-**	**-**	**-**	**-**	**-**	**-**	
**32**	**wild**	**A**	**7**	**M**	**-**	**-**	**-**	**-**	**-**	**-**	**1**	**-**	**-**	**-**	**2**	**55.5**
	**mut**	**A**	**7**	**A**	**-**	**-**	**-**	**-**	**-**	**-**	**1**	**-**	**-**	**-**	**1**	
**33**	**wild**	**A**	**9**	**Q**	**-**	**-**	**-**	**-**	**-**	**-**	**1**	**-**	**-**	**-**	**-**	**58.4**
	**mut**	**A**	**9**	**A**	**-**	**-**	**-**	**-**	**-**	**-**	**-**	**-**	**-**	**-**	**-**	
**34**	**wild**	**A**	**11**	**N**	**-**	**-**	**-**	**-**	**-**	**-**	**1**	**-**	**-**	**-**	**-**	**62.1**
	**mut**	**A**	**11**	**A**	**-**	**-**	**-**	**-**	**-**	**-**	**-**	**-**	**-**	**-**	**-**	
**35**	**wild**	**A**	**13**	**R**	**-**	**-**	**-**	**-**	**-**	**-**	**3**	**-**	**-**	**-**	**-**	**57.3**
	**mut**	**A**	**13**	**A**	**-**	**-**	**-**	**-**	**-**	**-**	**2**	**-**	**-**	**-**	**1**	
**36**	**wild**	**A**	**16**	**R**	**1**	**1**	**-**	**-**	**-**	**-**	**2**	**-**	**-**	**-**	**-**	**59.5**
	**mut**	**A**	**16**	**A**	**-**	**-**	**-**	**-**	**-**	**-**	**2**	**-**	**-**	**-**	**1**	
**37**	**wild**	**A**	**17**	**E**	**1**	**-**	**-**	**-**	**-**	**-**	**3**	**-**	**-**	**-**	**-**	**57**
	**mut**	**A**	**17**	**A**	**-**	**-**	**-**	**-**	**-**	**-**	**1**	**-**	**-**	**-**	**-**	
**38**	**wild**	**A**	**18**	**V**	**-**	**-**	**-**	**-**	**-**	**-**	**3**	**-**	**-**	**-**	**7**	**56.9**
	**mut**	**A**	**18**	**A**	**-**	**-**	**-**	**-**	**-**	**-**	**2**	**-**	**-**	**-**	**4**	
**39**	**wild**	**A**	**20**	**D**	**2**	**1**	**-**	**-**	**-**	**-**	**2**	**-**	**-**	**-**	**-**	**55.3**
	**mut**	**A**	**20**	**A**	**-**	**-**	**-**	**-**	**-**	**-**	**2**	**-**	**-**	**-**	**-**	
**40**	**wild**	**A**	**23**	**R**	**1**	**1**	**-**	**-**	**-**	**-**	**2**	**-**	**1**	**-**	**-**	**56.7**
	**mut**	**A**	**23**	**A**	**-**	**-**	**-**	**-**	**-**	**-**	**2**	**-**	**-**	**-**	**1**	
**41**	**wild**	**A**	**24**	**K**	**1**	**1**	**-**	**-**	**-**	**-**	**2**	**-**	**-**	**-**	**-**	**56.3**
	**mut**	**A**	**24**	**A**	**-**	**-**	**-**	**-**	**-**	**-**	**2**	**-**	**-**	**-**	**1**	
**42**	**wild**	**A**	**25**	**V**	**-**	**-**	**-**	**-**	**-**	**-**	**2**	**-**	**-**	**-**	**3**	**59.3**
	**mut**	**A**	**25**	**A**	**-**	**-**	**-**	**-**	**-**	**-**	**2**	**-**	**-**	**-**	**2**	
**43**	**wild**	**A**	**27**	**E**	**-**	**-**	**-**	**-**	**-**	**-**	**2**	**-**	**-**	**-**	**-**	**58.8**
	**mut**	**A**	**27**	**A**	**-**	**-**	**-**	**-**	**-**	**-**	**1**	**-**	**-**	**-**	**-**	
**44**	**wild**	**A**	**28**	**E**	**2**	**1**	**-**	**-**	**-**	**-**	**1**	**-**	**-**	**-**	**-**	**55.7**
	**mut**	**A**	**28**	**A**	**-**	**-**	**-**	**-**	**-**	**-**	**1**	**-**	**-**	**-**	**-**	
**45**	**wild**	**A**	**34**	**N**	**-**	**-**	**-**	**-**	**-**	**-**	**3**	**-**	**-**	**-**	**-**	**63**
	**mut**	**A**	**34**	**A**	**-**	**-**	**-**	**-**	**-**	**-**	**1**	**-**	**-**	**-**	**2**	

46	wild	A	35	S	-	-	-	-	-	-	2	-	-	-	-	63.4
	mut	A	35	A	-	-	-	-	-	-	2	-	-	-	-	
47	wild	A	39	Q	-	-	-	-	-	-	2	-	-	-	-	61.4
	mut	A	39	A	-	-	-	-	-	-	2	-	-	-	1	
48	wild	A	43	E	1	1	-	-	-	-	4	-	-	-	-	56.1
	mut	A	43	A	-	-	-	-	-	-	2	-	-	-	-	
49	wild	A	46	K	-	-	-	-	-	-	1	-	-	-	-	57.1
	mut	A	46	A	-	-	-	-	-	-	1	-	-	-	-	
50	wild	A	52	G	-	-	-	-	-	-	2	-	-	-	-	60.9
	mut	A	52	A	-	-	-	-	-	-	1	-	-	-	-	
Stable Mutations																
51	wild	A	8	P	-	-	-	-	-	-	1	-	-	-	2	74.1
	mut	A	8	A	-	-	-	-	-	-	1	-	-	-	1	
