# Supplementary material for: Engineering Proteins for Thermostability with iRDP Web Server
Source: PLoS One. 2015 Oct 5;10(10):e0139486. doi: 10.1371/journal.pone.0139486 (PMC4593602; doi:10.1371/journal.pone.0139486)
Supplement: S9 Table — (PDF) [file pone.0139486.s014.pdf]

S9 Table. iStability validation analysis showing prediction parameters calculated for different neutral-state energy cut-offs.

Threshold for Neutral-state (kcal/mol)	TP (True positive)	TN (True Negative)	FP (False Positive)	FN (False Negative)	FPR (False Positive Rate)	TPR (True Positive Rate)
0	47	8	9	17	0.53	0.84
0.1	46	8	9	18	0.53	0.84
0.2	45	9	8	19	0.47	0.85
0.3	45	10	7	19	0.41	0.87
0.4	41	11	6	23	0.35	0.87
0.5	39	11	6	25	0.35	0.87
0.6	36	12	5	28	0.29	0.88
0.7	33	12	5	31	0.29	0.87
0.8	33	12	5	31	0.29	0.87
0.9	30	12	5	34	0.29	0.86
1	29	13	4	35	0.24	0.88
1.5	18	14	3	46	0.18	0.86
2	9	15	2	55	0.12	0.82
2.5	5	16	1	59	0.06	0.83
3	3	16	1	61	0.06	0.75
3.5	2	16	1	62	0.06	0.67
4	1	16	1	63	0.06	0.50

A total of 81 mutations were analyzed for validation of four protein design strategies using iStability module with FoldX as stability prediction tool. For each mutation analyzed, FoldX gives energy scores (Table 2 and S7 Table) which represent the difference of stability between mutant and wild-type structure. A positive FoldX energy score usually indicates the decrease of stability upon mutation (D-state) while a negative score indicates increase of stability upon mutation (I-state). It is important to decide margin of energy scores between which a neutral state (N-state) can be predicted. By using different FoldX energy score cut-offs, neutral states were defined and for each N-state categories, predictions were compared with experimental observation and ROC curve was generated.

1. When no neutral-state cut-off was used, 2 states of predictions obtained
    a. I-state: FoldX Energy score < 0
    b. D-state: FoldX Energy score > 0
2. For neutral-state cutoff of **0.1**, 3 states of predictions obtained
    a. I: FoldX Energy score < -0.1
    b. N: FoldX Energy score between -0.1 to 0.1
    c. D: FoldX Energy score > 0.1
3. For neutral-state cutoff of **0.2**, 3 states of predictions obtained
    a. I: FoldX Energy score < -0.2
    b. N: FoldX Energy score between -0.2 to 0.2
    c. D: FoldX Energy score > 0.2

Similarly different neutral-state cut-offs (**0, 0.1, 0.2, 0.3, 0.4, 0.5, 0.6, 0.7, 0.8, 0.9, 1, 1.5, 2, 2.5, 3, 3.5 and 4**) were used, and True-positive rate (TPR) and False-positive rate (FPR) was calculated. ROC curve was then generated.

- **True Positive (TP):** Both experiment and prediction showed increase (I) of stability.
- **False Positive (FP):** Prediction showed increase (I) of stability while experiment showed either decrease/no-change (D/N) in stability.
- **True negative (TN):** Both experiment and prediction showed decrease/no-change (D/N) in stability.
- **False negative (FN):** Experiment showed increase (I) of stability while prediction showed either decrease/no-change (D/N) in stability.
